# Deciphering the membrane topology of the pestiviral non-structural protein 4B (NS4B)

**DOI:** 10.1128/jvi.00825-25

**Published:** 2025-08-13

**Authors:** S. Höppner, O. Isken, N. Tautz

**Affiliations:** 1 Institute of Virology and Cell Biology, University of Lübeck 237099, Lübeck, Germany; University of Michigan Medical School, Ann Arbor, Michigan, USA

**Keywords:** BVDV, *Pestivirus*, *Flaviviridae*, NS4B, positive-strand RNA virus, membrane topology

## Abstract

Pestiviruses like bovine viral diarrhea virus and other members of the family *Flaviviridae* form replication complexes consisting of viral non-structural and cellular proteins at rearranged intracellular membranes. Despite the pivotal roles of non-structural protein 4B (NS4B) throughout the pestiviral life cycle, little is known about how this protein exerts its multiple functions. It is assumed that pestiviral NS4B promotes replication complex assembly and virion morphogenesis by interacting with defined sets of viral and host proteins. The membrane topology of the protein dictates the availability of individual protein structures and interfaces for such interactions. Thus, the knowledge of the NS4B membrane topology is required for a detailed functional understanding of this protein. Therefore, we experimentally determined the membrane topology for NS4B *in cellulo* by using the substituted cysteine accessibility method (SCAM) in combination with computational secondary structure and transmembrane domain (TMD) predictions. Our model indicates the formation of two TMDs in the N-terminal region of NS4B (TMD2–3) followed by nine putative membrane-associated α-helices. Furthermore, a dual topology of the N-terminal amphipathic α-helix AH1 was detected by applying a Split-GFP assay, exposing similarities to hepatitis C virus NS4B. The translocation of AH1 across the membrane and the luminal orientation of the proposed loop connecting TMD2–3 was further confirmed by glycosylation acceptor site recognition analysis. Together, our model will assist further studies on the diverse functions of pestiviral NS4B throughout the viral life cycle.

**IMPORTANCE:**

Membrane proteins are of special importance for positive-strand RNA viruses due to their replication at remodeled intracellular membranes. Moreover, the multi-functionality of these proteins can rely on alternative topologies. Studying their membrane topologies is challenging since protein purification can induce misfolding. Similarly, random insertions of large N-glycosylation acceptor sites may disturb transmembrane domains and thus the topology, while minimal glycosylation motifs (NXT/S) are often inefficiently glycosylated. Therefore, we used the SCAM assay utilizing single cysteine substitutions to analyze the membrane topology of BVDV-1 NS4B. A dual topology of the N-terminal region was demonstrated by a Split-GFP assay. Glycosylation acceptor site insertions at pre-analyzed positions further corroborated the model. In sum, BVDV-1 NS4B topology shows similarities but also remarkable differences to the NS4B membrane topologies of other *Flaviviridae* orthologues. This new information will allow further studies to clarify the molecular basis of the multi-functionality of this critical viral component.

## INTRODUCTION

Alongside classical swine fever virus (CSFV) and border disease virus (BDV), bovine viral diarrhea viruses 1 and 2 (BVDV-1 and -2) are classified as species *Pestivirus suis, Pestivirus ovis, Pestivirus bovis*, and *Pestivirus tauri*, respectively, in the genus *Pestivirus* within the family *Flaviviridae* (1). With their broad host range, including swine and ruminant hosts, these animal pathogens represent a significant burden to livestock farming worldwide (2). Two biotypes of BVDV, cytopathogenic (cp) and non-cytopathogenic (ncp), are recognized based on their phenotype in cultured epithelial cells. The cp biotype does not correlate with virulence in acute infections as BVDV strains associated with severe acute BVD outbreaks are all non-cytopathogenic (3). The enveloped viral particles contain a positive-sense single-stranded RNA genome with an approximated size of 12.3 kb. The encoded open reading frame (ORF) is flanked by 5´ and 3´ untranslated regions comprising regulatory elements for translation and RNA replication (3–5). The polyprotein is co- and post-translationally cleaved by cellular and viral proteases into the mature structural proteins (SPs) Core, E^rns^, E1, and E2, which are present in the virions, and the non-structural proteins (NSPs) N^pro^, p7, NS2, NS3, NS4A, NS4B, NS5A, and NS5B, which serve pivotal functions during viral RNA replication and virion morphogenesis (6). The limited genomic coding capacity requires a temporal orchestration of polyprotein cleavage to form various intermediate precursor products and the formation of different multi-protein complexes during the individual stages of the pestiviral life cycle (7–11). NS4B is part of the minimal replication complex (replicase) formed by NS3 to NS5B together with an undefined set of cellular factors and plays a crucial yet poorly understood role in viral RNA synthesis (9, 12–14). It is assumed that genome replication of BVDV occurs in close association with intracellular membranes, as described for other positive-strand RNA viruses. In the case of the hepatitis C virus (HCV), a closely related member of the family *Flaviviridae*, infected cells contain accumulations of vesicles forming a membranous web (MW) that is thought to be the site of viral RNA replication. For HCV, it was shown that NS3, NS4A, NS4B, NS5A, and NS5B assemble the viral replicase on the endoplasmatic reticulum (ER) forming double-membrane vesicles (DMVs) as part of the MW (15). However, the knowledge about the underlying molecular mechanisms is still incomplete. NS4B of HCV is a multi-spanning integral membrane protein, and its oligomerization is required for viral replicase assembly (16). For BVDV, an interaction between NS3, NS4B, and NS5A was shown, similar to what has been described for HCV (16–18). BVDV NS4B is also a hydrophobic protein integrated into intracellular membranes (19). It is assumed that the biochemical properties of BVDV NS4B allow this protein to serve as a membrane anchor for the viral replication complex at ER-derived membranes similar to HCV (15, 20, 21). Single- and double-membrane vesicles characterize the membranous web induced by HCV (15, 21). The appearance of DMVs correlates with active RNA replication, and their induction depends on NS5A, whereas NS4B can induce single-membrane vesicles. This observation is arguing that MW formation requires the concerted action of several HCV replicase proteins (22). While highly likely, a comparable membrane remodeling connected to pestiviral RNA replication was so far not unequivocally demonstrated. However, membrane rearrangements have been reported in BVDV-infected cells (19). Nevertheless, there is mounting evidence that the pestiviral life cycle also obliges the association to the ER membrane (23, 24). An impact of pestiviral NS4B on the viral biotype was postulated due to a biotype switch of a BVDV-1 strain from cp to ncp based on a single mutation at residue 15 of NS4B (17). With regard to sequence similarities of NS4B, nucleotide-binding motifs, designated as Walker A and Walker B, respectively, were identified in pestiviral and hepaciviral isolates with differences in the location of these motifs. In CSFV, these motifs were associated with binding and hydrolysis of ATP and GTP. In the case of HCV NS4B, an in-depth biochemical characterization demonstrated that this protein binds both ATP and GTP but has a higher affinity for ATP, while an NS4B-GFP (green fluorescent protein) fusion protein was found to bind GTP preferentially in transfected Huh-7 cells (25, 26). Nevertheless, mutagenesis of the motif sequences had no impact on the viral replication or virion morphogenesis in CSFV, whereas HCV replication was strongly impaired (26, 27). In addition, other regions of NS4B were reported to modulate HCV RNA replication. The disruption of the hydrophobic interface of NS4B AH1 was shown to abolish viral RNA synthesis with effects on the biogenesis of DMVs and mislocalization of the replication complex (28, 29). Moreover, the interaction between the C-terminal NS4B and the N-terminal NS5A region is crucial for proper NS5A localization with consequences for RNA replication upon mutagenesis (30). The functionally important HCV NS4B oligomerization was reported to be mediated via the N-terminal (AH1/AH2) (16, 31) and via the C-terminal region, possibly with contributions of a C-terminal palmitoylation (16, 20, 32). Furthermore, there is evidence from dengue virus (DENV) and West Nile virus that orthoflaviviral NS4B dimerizes via their C-terminal region (33). These data on the involvement of orthoflaviviral and hepaciviral NS4B in viral replication emphasize the importance of the protein’s membrane topology for providing potential interaction platforms for other proteins. The current membrane topology model of hepaciviral NS4B proposes the formation of the N-terminal amphipathic α-helices AH1/AH2 located in the cytosol (28, 34), followed by four transmembrane domains (TMD1–4) (35) and the cytoplasmic α-helices H1/H2 at the C terminus (36, 37). To allow for polyprotein processing by the NS3/4A protease, the N- and C-terminal parts are believed to be located on the cytosolic side of the ER membrane. Intriguingly, further studies demonstrated the partial translocation of the N terminus post-translationally to the ER lumen, which is conserved between all genotypes of HCV and was supposed to influence DMV formation (34, 38). Specifically, it was shown that an amphipathic helix (aa 42 to 66) has the potential to traverse the phospholipid bilayer as a transmembrane segment, likely upon oligomerization (34). On the contrary, distinct NS4B membrane topology models were described for different *Orthoflavivirus* species despite similar computational predictions that deviate from the proposed hepaciviral model (39). Taken together, there are differences with regard to NS4B membrane topology of various members of the *Flaviviridae* family. Importantly, there is no membrane topology model for pestiviral NS4B described yet in the literature, and its function in the viral life cycle is still incompletely understood. Here, we applied the substituted cysteine accessibility method (SCAM) (40) to determine the membrane topology of the pestiviral NS4B in combination with computational predictions of its secondary structure and TMD formation. Our studies revealed that the membrane topology of pestiviral NS4B shows similarities but also marked differences to the HCV NS4B model.

## MATERIALS AND METHODS

### Multiple sequence alignment and secondary structure prediction of pestiviral NS4B

Multiple sequence alignment of the protein sequence of NS4B was performed using ClustalX2 (41) with the following pestiviral strains (GenBank accession number in parentheses): *Pestivirus bovis*—BVDV-1 CP7 (AAC55984.1) and NADL (AIE38087.1), *Pestivirus tauris*—BVDV-2 890 (AAA82981.1), *Pestivirus suis*—CSFV Alfort/187 (CAA61161.1), *Pestivirus ovis*—BDV X818 (AAC16444.1), *Pestivirus australiaense*—porcine pestivirus Bungowannah (ABO21120.2), *Pestivirus giraffae*—giraffe pestivirus H138 (AAF02523.2), *Pestivirus braziliense*—HoBi-like pestivirus Th/4_KhonKaen (ACM79934.1), and *Pestivirus aydinense*—Aydin-like pestivirus 04-TR (AFS63897.1). The corresponding phylogenetic tree was visualized with the online application “Interactive Tree of Life” (https://itol.embl.de) (42). The prediction of the protein secondary structure for the individual species listed above was performed using the web server “Proteus” (http://www.proteus2.ca/proteus) (43). Putative helical elements were analyzed for their hydrophobicity and hydrophobic moment with HeliQuest (https://heliquest.ipmc.cnrs.fr/index.html) (44).

### Prediction of transmembrane domains

The potential formation of transmembrane regions in NS4B was analyzed for BVDV-1 CP7 using the online tools as follows: HMMTOP (http://www.enzim.hu/hmmtop/) (45), DeepTMHMM (https://dtu.biolib.com/DeepTMHMM) (46), TOPCONS (https://topcons.cbr.su.se/) (47), PolyPhobius (https://phobius.sbc.su.se/poly.html) (48), SPLIT (http://pref.etfos.hr) (49), MEMSAT-SVM (http://bioinf.cs.ucl.ac.uk/psipred/) (50), PredictProtein (https://predictprotein.org/) (51), and MemBrain (http://www.csbio.sjtu.edu.cn/bioinf/MemBrain/) (52, 53).

### Tertiary structure prediction

The spatial structure of BVDV-1 CP7 NS4B was assessed using AlphaFold v.2.3.2 (54) and v.3 (55) with default settings and the following adjustments (only for v.2.3.2). The models were calculated from one random seed using MSA mode “mmseqs2_uniref_env,” 20 recycles, and pair mode “paired” with template structures fetched from the Protein Data Bank (PDB) 100. The resulting models were evaluated using ChimeraX v.1.9 (56).

### Antibodies

BVDV NS3/NS2-3 were detected by mouse monoclonal antibody (mAb) 8.12.7, kindly provided by E. J. Dubovi (Cornell University, Ithaca, NY) (57). The mouse mAb directed against NS5A (GLBVD5A1 [11c]) was kindly provided by T. Rümenapf (University of Veterinary Medicine, Vienna, Austria) and B. Lamp (Justus Liebig University, Giessen, Germany) (58). All virus-specific mAbs were used as unpurified hybridoma supernatants. The HA epitope was detected using either the rabbit α-HA mAb C29F4 or the mouse α-HA mAb 6E2, both purchased from Cell Signaling Technology Europe (Leiden, The Netherlands). Species-specific peroxidase-coupled secondary antibodies and the goat α-mouse IgG-specific Cy3-labeled secondary antibody were obtained from Dianova (Hamburg, Germany). The goat α-rabbit IgG-specific AlexaFluor488-labeled secondary antibody was obtained from Invitrogen/Thermo Fisher Scientific (Schwerte, Germany).

### Cells and viruses

Madin-Darby bovine kidney (MDBK) and human embryonic kidney (HEK) 293T cells were obtained from American Type Culture Collection (Manassa, VA, USA). MDBK cells were cultivated in Dulbecco’s modified Eagle’s medium (DMEM) supplemented with 10% horse serum (Thermo Fisher Scientific, Schwerte, Germany), 100 U/mL penicillin, and 100 µg/mL streptomycin (Sigma-Aldrich, Taufkirchen, Germany). HEK293T cells and Huh7-T7 (59) cells were grown in DMEM containing 10% fetal calf serum (Thermo Fisher Scientific, Schwerte, Germany), 100 U/mL penicillin, and 100 µg/mL streptomycin. For the latter, the culture medium was additionally supplemented with 125 µg/mL G418 (Thermo Fisher Scientific, Schwerte, Germany). All cells were grown at 37°C and 5% CO_2_. The BVDV-1 strain NCP7 was described previously (60, 61). The modified vaccinia virus Ankara (MVA)-T7^pol^ was generously provided by G. Sutter (LMU, Munich, Germany) (62).

### Plasmid constructs

Plasmids were generated by standard cloning techniques. Mutations were introduced by PCR or QuikChange site-directed mutagenesis kit (Thermo Fisher Scientific, Schwerte, Germany). All constructs were verified by restriction enzyme digest and sequencing. PCR products were subcloned using the pGEM-T vector system I (Promega, Madison, WI, USA). Restriction enzymes used were purchased from New England Biolabs (NEB, Frankfurt/Main, Germany). All amino acid numbers refer to the individual sequence of the respective protein encoded by BVDV-1 CP7 (GenBank accession number AAC55984.1). Lowercase letters in PCR primer sequences represent sequences complementary to the template, while uppercase letters indicate insertions or modifications. Recognition sites of restriction enzymes used for cloning are underlined.

The insertion of a HA-epitope tag after amino acid 38 and of the double-tag StrepHA at the C terminus of NS4B from BVDV-1 strain NCP7, respectively, for detection in Western blot (WB) and immunofluorescence was performed as described below. First, the recognition sites for *Mlu*I and *Asc*I were introduced into the subclone vector pSK-BVDV-SacI-SalI (*Sac*I-*Sal*I fragment derived from the pNCP7-388 plasmid encoding the C-terminal part of NS3, NS4A, and the N-terminal part of NS4B) (63) for the HA tag and into the subclone vector pKS-BVDV-SalI-ClaI (*Sal*I-*Cla*I fragment derived from the pNCP7-388 plasmid [60, 61] encoding the C-terminal part of NS4B) by QuikChange mutagenesis after aa 38 (5′-gagatctgcccccgctttcaaagaaacgcgtCCGCggcgcgccAaacgtggaagctgctaaagggtacg-3′), thus generating pKS-BVDV-SacI-SalI/MluI-AscI, and at the C terminus (5′-gaagataaggaacctgacgcgtTATGggcgcgccAtctgggaattatgtcctg), thereby generating pKS-BVDV-SalI-ClaI/MluI-AscI, respectively. Next, the oligonucleotides HA-tag sense (5′-cgcgttacccctatgacgtgccagactacgct-3′) and HA-tag ase (5′-cgcgccagcgtagtctggcacgtcataggggta-3′) as well as StrepHA sense (5′-cgcgtagtggttggagtcacccccagtttgagaaggggtccggatacccgtacgatgttccggattatgcttcctat-3′) and StrepHA ase (5′-cgcgccataggaagcataatccggaacatcgtacgggtatccggaccccttctcaaactgggggtgactccaaccact-3′) were phosphorylated at the 5′-end for 30 min at 37°C using a T4 polynucleotide kinase (Thermo Fisher Scientific, Schwerte, Germany) followed by a 5 min denaturation at 95°C and a gradual cool-down to room temperature with a 5°C/min increment. The double-stranded oligonucleotides were incorporated into the subclone vector pKS-BVDV-SacI-SalI/MluI-AscI for the HA tag (resulting in pSK-BVDV-SalI-SacI/4B-38HA) and into the subclone vector pKS-BVDV-SalI-ClaI/MluI-AscI for the StrepHA tag via *Mlu*I and *Asc*I (pKS-BVDV-SalI-ClaI/4B-CT-StrepHA). To maintain an authentic NS4B C terminus, a repeat of the last five amino acids of the protein with altered genetic code to prevent RNA recombination (5′-gttatgggcgcgccaAAAATTCGTAATTTAtctgggaattatgtcctg-3′) was integrated into the pKS-BVDV-SalI-ClaI/4B-CT-StrepHA vector in a consecutive reaction, generating pKS-BVDV-SalI-ClaI/4B-CT-StrepHA-5aa.

The subclone vectors pSK-BVDV-SalI-SacI/4B-38HA and pKS-BVDV-SalI-ClaI/4B-CT StrepHA-5aa were used to generate the expression plasmids pCITE-Ubi-NS3-5B/4B-38HA and pCITE-Ubi-NS3-5B/4B-CT-StrepHA-5aa as well as the full-length cDNA clones pNCP7-388-Rluc/4B-38HA and pNCP7-388-Rluc/4B-CT-StrepHA-5aa by using the *Sac*I-*Sal*I or *Sal*I-*Cla*I restriction sites with pCITE-Ubi-NS3-5B (63) or pNCP7-388-Rluc (64) as vector, respectively.

The expression plasmids pCITE-NS4B-38HA and pCITE-NS4B-CT-StrepHA-5aa were constructed as follows. The encoding regions of NS4B-38HA and NS4B-CT-StrepHA-5aa were amplified from pCITE-Ubi-NS3-5B/4B-38HA and pCITE-Ubi-NS3-5B/4B-CT-StrepHA-5aa vectors with the primers CITE-NcoI sense (5′-ccatggcagtgggtgacttg-3′) and NS4B-WT-XbaI ase (5′-tctagaTTAcaggttccttatcttccc-3′) for pCITE-NS4B-38HA or NS4B-CT-5aa-NotI ase (5′-gcggccgcTTAtaaattacgaatttttggcgcgcc-3′) for pCITE-NS4B-CT-StrepHA-5aa, respectively. The fragments were gel-purified and subcloned into the pGEM-T vector (resulting in pGEM-T NS4B-38HA and pGEM-T NS4B-CT-StrepHA-5aa, respectively). pCITE-NS4B-38HA and pCITE-NS4B-CT-StrepHA-5aa were generated by ligation of NS4B-38HA and NS4B-CT-StrepHA-5aa fragments from pGEM-T NS4B-38HA and pGEM-T NS4B-CT-StrepHA-5aa into pCITE-2a (Novagen, Madison, WI, USA) via *Nco*I and *Not*I.

The NS4B cysteine mutants were generated using QuikChange mutagenesis with the template pCITE-NS4B-CT-StrepHA-5aa for modification of NS4B amino acids prior to aa 300 and pCITE-NS4B-38HA for changes of NS4B amino acids from aa 300 to the C terminus. A list of the sequences of the primers used for site-directed mutagenesis is available on request.

The expression constructs pCITE-NS4B-EC4-CT-StrepHA-5aa were designed as follows. The encoding sequence of the extracellular loop 4 (EC4) of the human erythrocyte anion exchanger-1 (QKLSVPDGFKVS**NSS**ARGWVIHPLGLRS) (65) was fused to the N terminus and introduced into NS4B by QuikChange mutagenesis with pCITE-NS4B-CT-StrepHA-5aa as template, thereby generating pCITE-EC4-NS4B-CT-StrepHA-5aa. For the C-terminal fusion of NS4B with EC4, pCITE-NS4B-38HA was used as template, resulting in pCITE-NS4B-38HA-EC4. The incorporated glycosylation motif is highlighted in bold. A list of the sequences of the used primers is available on request.

For the construction of NS3-5B expression plasmids encoding the N-terminal EC4-NS4B fusion protein, overlap PCR was used. To ensure intact polyprotein processing, upstream of the N-terminal EC4 loop, a repeat of the first 5 aa of NS4B was incorporated. First, the fragment NS3(433)-4A (encoding the aa 433-683 of NS3 and NS4A) was amplified with pCITE-Ubi-NS3-5B (63) as template using the primers BVDV6500 sense (5′-gacccagctaacttgagagtgg-3′) and NS4A ase (5′-acggacagcttctgCAAGTCACCCACTGC-3′). Second, the fragment NT-EC4-NS4B(324) (encoding the EC4-NS4B until NS4B aa 324) was generated by PCR with the primers NS4B sense (5′-GCAGTGGGTGACTTGcagaagctgtccgt-3′) and NS4B-324 ase (5′-gtccagacaattggcaaccc-3′) with pCITE-EC4-NS4B-CT-StrepHA-5aa as template. The overlapping sequence is highlighted in capitals in the primer sequence. Afterwards, the gel-purified fragments NS3(433)-4A and NT-EC4-NS4B(324) were joined by overlap PCR to generate NS3(433)-4A-NT-EC4-NS4B(324) for 15 cycles at 60°C annealing temperature using 1:10 dilutions of the fragments followed by PCR amplification of the joint fragment using the primers BVDV6500 sense and NS4B-324 ase. The gel-purified fragment NS3(433)-4A-NT-EC4-NS4B(324) was subcloned into the pGEM-T vector [resulting in pGEM-T NS3(433)-4A-NT-EC4-NS4B(324)]. The expression vector pCITE-Ubi-NS3-5B/NT-EC4-4B-CT-StrepHA-5aa was generated by ligation of the NS3(433)-4A-NT-EC4-NS4B(324) fragment from pGEM-T NS3(433)-4A-NT-EC4-NS4B(324) into pCITE-Ubi-NS3-5B/4B-CT-StrepHA-5aa via *Blp*I and *Sal*I.

The bicistronic expression plasmids pWPI-GFP(1-10) and pWPI-GFP(1-10)-KDEL encoding in the second ORF either GFP11-NS4B-CT-StrepHA-5aa or HA-NS4B-GFP11 were cloned as described below. Initially, the restriction site for *BamH*I was inserted by QuikChange mutagenesis into the plasmid pWPI-msc-BLR (66) downstream of the blasticidin resistance gene (BLR) to enable alterations of the second ORF of the vector. Therefore, the primer pair pWPI-BLR(+BamHI) (5′-gctaactcgaggGGATCCcgataatcaacctc-3′) was used with the subclone pSK-KpnI-BLR-EagI as template, which contained the *Kpn*I-*Eag*I fragment derived from pWPI-mcs-BLR, including the blasticidin resistance gene and surrounding sequences. This plasmid was generated by digesting pWPI-mcs-BLR with *Kpn*I and *Eag*I followed by ligation of the gel-purified BLR-encoding fragment into pBluescript SK- (Stratagene/Agilent Technologies, Santa Clara, CA, USA). To generate a GFP(1-10) protein localized to the ER, the encoding sequence of GFP(1-10) was amplified from the template pcDNA3.1-GFP(1-10) (67) with addition of an N-terminal ER signal peptide (MGWSCIILFLVATATGAHS) and a C-terminal ER retention signal (SEKDEL) as previously described by Hyun et al. (68) using the primers BamHI/SP/GFP(1-10) sense (5′-GGATCCATGGGATGGAGCTGTATCATCCTCTTCTTGGTAGCAACAGCTACAGGCGCGCACTCCtccaaaggagaagaactgtttac-3′) and GFP(1-10)/SEKDEL/XbaI ase (5′-TCTAGACTACAATTCGTCCTTTTCGCTtttttcatttggatctttgctcag-3′). The fragment was subcloned into the pGEM-T vector followed by ligation into pcDNA3.1 (Thermo Fisher Scientific, Schwerte, Germany) via *BamH*I and *Xba*I. For insertion of GFP(1-10) or GFP(1-10)-KDEL into the multiple cloning site (mcs) of pWPI-mcs-BLR, the corresponding encoding sequence was amplified from the pcDNA3.1 vectors mentioned above as templates with the primers SbfI-GFP(1-10) sense (5′-TACCCTGCAGGATGtccaaaggagaagaac-3′ and MluI-GFP(1-10) ase (5′-TACGACGCGTTCAtttttcatttggatctttg-3′), or SbfI-GFP(1-10)-KDEL sense (5′-TACCCTGCAGGATGggatggagctgtatc-3′) and MluI-GFP(1-10)-KDEL ase (5′-TACGACGCGTTCAcaattcgtccttttcgc-3′), respectively. After gel purification, the fragments were ligated into pWPI-mcs-BLR via *Sbf*I and *Mlu*I, generating pWPI-GFP(1-10) and pWPI-GFP(1-10)-KDEL, respectively. The GFP11 peptide (RDHMVLHEYVNAAGIT) was inserted together with a GS linker (GGSGGGS) at the N terminus of NS4B into pCITE-NS4B-CT-StrepHA-5aa by QuikChange mutagenesis (5′-acgatgataataccatgCGGGACCACATGGTGCTGCACGAGTACGTGAACGCCGCC

GGCATCACAGGCGGCAGCGGCGGCGGCAGCgcagtgggtgacttgg-3′), resulting in pCITE-GFP11-NS4B-CT-StrepHA-5aa. For the C-terminal fusion, two consecutive reactions were performed. First, a HA-epitope tag was introduced at the N terminus of NS4B by amplification of the encoding sequence from pCITE-NS4B CT-StrepHA-5aa with the primer pair HA-NS4B sense (5′-ccatggCGTACCCGTACGATGTTCCGGATTATGCTgcagtgggtgacttggac-3′) and ase (5′-CTCGAGTCAcaggttccttatcttcccttc 3′). The fragment was subcloned into the pGEM-T vector and then ligated into pCITE-2a via *Nco*I and *Xho*I, generating pCITE-HA-NS4B. Second, the GFP11 peptide with the GS linker was inserted at the C terminus of NS4B into pCITE-HA-NS4B by QuikChange mutagenesis (5′-gaagataaggaacctgGGCGGCAGCGGCGGCGGCAGCCGGGACCACATGGTGCTGCACGAGTACGTGAACGCCGCCGGCATCACAtgactcgagcaccacc-3′) to obtain pCITE-HA-NS4B-GFP11. Subsequently, the BLR gene in pWPI-GFP(1-10) and pWPI-GFP(1-10)-KDEL was exchanged via *Kpn*I and *BamH*I with GFP11-NS4B-CT-StrepHA-5aa or HA-NS4B-GFP11, generating pWPI-GFP(1-10)-IRES-GFP11-NS4B-CT-StrepHA-5aa and pWPI-GFP(1-10)-KDEL-IRES-GFP11-NS4B-CT-StrepHA-5aa as well as pWPI-GFP(1-10)-IRES-HA-NS4B-GFP11HA and pWPI-GFP(1-10)-KDEL-IRES-HA-NS4B-GFP11 after their amplification from pCITE-GFP11-NS4B-CT-StrepHA-5aa and pCITE-HA-NS4B-GFP11, respectively, using the primer cite100 sense (5′-caaaggaatgcaaggtctgttg-3′), and GFP11-NS4B/BamHI ase (5′-TATGGATCCTTAtaaattacgaatttttggcgc-3′) or NS4B-GFP11/BamHI ase (5′-TATGGATCCTCATGTGATGCCGGCG-3′), respectively.

### *In vitro* transcription and RNA electroporation

Full-length BVDV-1 NCP7-388-Rluc RNA was electroporated into MDBK cells as previously described (69). *In vitro* transcription was performed with a HiScribe SP6 transcription kit (NEB, Frankfurt/Main, Germany) using 1.5 µg template DNA linearized with *Sma*I (NEB, Frankfurt/Main, Germany) and purified by phenol/chloroform extraction. The RNA quality was verified by non-denaturing agarose gel electrophoresis, and 1 µg RNA was used for electroporation of 3 × 10^6^ MDBK cells (conditions: 180 V, 950 µF, 2 mm-gap cuvette) using a GenePulserII electroporation device (Biorad, Munich, Germany). Cells were immediately transferred into supplemented DMEM and seeded as required into six-well plates. At defined time points post-electroporation (p.e.), cells were harvested for luciferase assay or fixated for immunofluorescence staining.

### DNA transfection and transient protein expression

For transient protein expression by EF1α promoter-regulated gene cassettes, HEK293T cells were used, while Huh7-T7 cells served for T7 polymerase-driven gene expression. For the latter, the cytoplasmic level of T7 RNA polymerase (T7^pol^) of Huh7-T7 cells was increased prior to DNA transfection by infection with a modified vaccinia virus Ankara encoding T7^pol^ (MVA-T7^pol^) (62) for 1 h at 37°C. The HEK293T or MVA-T7^pol^-infected Huh7-T7 cells were transfected with 4 µg–8 µg of plasmid DNA using polyethylenimine (Polysciences, Inc., Warrington, PA, USA) as transfection reagent. Protein expression was carried out for 20–24 h at 37°C, and the cells were either directly processed for SDS-PAGE, fixated for immunofluorescence staining, or used in cell-based assays, respectively.

### SDS-PAGE and Western blotting

The cells of one well of a six-well plate were lysed in 50 µL of cold lysis buffer (0.5% [vol/vol] *n*-dodecyl-β-d-maltoside [Thermo Fisher Scientific, Schwerte, Germany]; 100 mM NaCl; 20 mM Tris; pH 7.5) for 10 min at 4°C followed by centrifugation for 10 min at 15,700 × *g* at 4°C. The supernatant was mixed with 50 µL of SDS sample buffer and incubated at 95°C for 10 min. A total of 20 µL of each sample was separated by SDS-polyacrylamide-tricine gel electrophoresis (70), subsequently transferred onto nitrocellulose membranes (Pall, Pensacola, FL, USA), followed by blocking of the membranes with 5% (wt/vol) skim milk powder (Roth, Karlsruhe, Germany) in phosphate buffered saline (PBS) with 0.05% (vol/vol) Tween 20 (Serva, Heidelberg, Germany). Proteins were detected with the indicated monoclonal antibodies and species-specific peroxidase-coupled secondary antibodies. Peroxidase activity was visualized using the Western Lightning Chemiluminescence Reagent (PerkinElmer, Boston, MA, USA) at the ImageQuant LAS-4000 mini-camera system (Fujifilm, Duesseldorf, Germany).

### Luciferase assay

The replication efficiency of viral BVDV-1 NCP7-388-Rluc RNA genomes was determined in correlation to the activity of *Renilla* luciferase with the *Renilla* Glow-Juice kit (PJK, Kleinblittersdorf, Germany) according to the manufacturer’s protocol. For this, full-length cDNA clones of BVDV-1 NCP7-388 with a Rluc-Ubi insertion between N^pro^ and core (64) were applied. After electroporation or infection, cells were harvested at 2 h, 48 h, and 72 h p.e. or 72 h post-infection (p.i.), respectively, and stored at −80°C. The samples were lysed in 40 µL lysis juice for 10 min at 4°C and precleared by centrifugation at 15,700 × *g* for 1 min. A total of 30 µL lysate was added to 100 µL of *Renilla* Glow-Juice containing 2 µL of coelenterazine substrate solution, and luciferase activity was measured with a portable tube luminometer (model Junior LB9509; Berthold, Bad Wildbad, Germany) and reported as the number of relative light units (RLUs).

### BVDV infection

The cell culture supernatant of MDBK cells electroporated with BVDV-1 NCP7-388-Rluc RNA was collected 72 h p.e., filtered through a 0.2 µm cellulose filter (Sartorius, Goettingen, Germany), and 500 µL of the cell culture supernatant was used for infection of 4 × 10^5^ naïve MDBK cells at 37°C in supplemented DMEM. Viral infection was determined 72 h p.i. by a luciferase assay or immunofluorescence staining with mAb 8.12.7 directed against NS3/NS2-3 combined with a goat α-mouse IgG-specific Cy3-labeled secondary antibody (Dianova, Hamburg, Germany).

### Nucleotide sequencing

DNA sequencing was performed by the sequencing service at LGC Genomics GmbH (Berlin, Germany). Sequence analysis was done with the online application Benchling (https://benchling.com).

### Immunofluorescence analysis

Protein expression and virus infection were detected by indirect immunofluorescence using the indicated mAb together with fluorophore-coupled IgG-specific secondary antibodies. For this purpose, cells were fixated with 2% (wt/vol) paraformaldehyde (PFA) (Thermo Fisher Scientific, Schwerte, Germany) in PBS for >20 min at 4°C. Afterwards, the cells were permeabilized with 0.5% (wt/vol) *N*-octyl-β-D-glycopyranoside (Sigma-Aldrich, Taufkirchen, Germany) in PBS for 10 min at 4°C, followed by two washing steps with PBS. Then, the cells were incubated for 30 min in blocking solution (0.05% [vol/vol] Tween-20 and 2% [wt/vol] bovine serum albumin [Serva, Heidelberg, Germany] in PBS). Subsequently, the cells were incubated for 1 h at 37°C with primary antibodies diluted in blocking solution (as noted in parentheses): mouse α-NS3/NS2-3 mAb 8.12.7 (1:40); rabbit α-HA mAb C29F4 (1:1,000). After washing two times with 0.05% (vol/vol) Tween-20 in PBS (PBS-T), the cells were incubated for 45 min at 37°C with fluorophore-coupled, species IgG-specific antibodies diluted in blocking solution (as described in parentheses): goat α-mouse IgG mAb-Cy3 (1:2,000); goat α-rabbit IgG mAb-Alexa488 (1:500). Cellular nuclei were simultaneously stained using DAPI (4´,6-diamidino-2-phenylindole, 2 mg/mL) diluted 1:3,000 in blocking solution. Finally, cells were washed with PBS-T and overlaid with PBS. Images were obtained with a Zeiss Axio Observer Z1 fluorescence microscope (Zeiss, Goettingen, Germany) equipped with the acA5472-5gm GigE camera (Basler, Ahrensburg, Germany). Captured images were processed using ImageJ software (NIH, Bethesda, MD, USA).

### SCAM

Expression plasmids of the NS4B cysteine derivatives were generated by site-directed mutagenesis as mentioned above. SCAM was performed as previously described (40, 71). The proteins were transiently expressed in MVA-T7^pol^-infected Huh7-T7 cells after DNA transfection and equal cell amounts lysed with either 2% (vol/vol) Triton X-100 (Fluka/Honeywell, Charlotte, NC, USA) or 0.15% digitonin (Carl Roth, Karlsruhe, Germany), respectively, for 10 min at room temperature (RT). Subsequently, one-half of each cell suspension was treated with 2 mM methoxy polyethylene glycol-5000-maleimide (PEG-maleimide, 5 kDa) (Sigma-Aldrich, Taufkirchen, Germany) for 30 min at RT. The reactions were stopped by addition of 50 mM dithiothreitol (DTT) (Carl Roth, Karlsruhe, Germany) followed by a 10 min incubation on ice. To remove the reaction compounds, proteins were purified by acetone precipitation as follows. The samples were mixed with 2.5 volumes of ice-cold acetone by shaking vigorously and incubated for 2 h or overnight at −20°C. Proteins were pelleted by centrifugation at 15,700 × *g* and 4°C for 30 min followed by one washing step with 70% (vol/vol) ethanol. The protein pellets were dried at RT for <30 min, solubilized in 100 µL SDS sample buffer, and 20 µL of each sample was separated by SDS-PAGE using 10% polyacrylamide-tricine gels (70). Afterwards, proteins were transferred to nitrocellulose membranes and detected using the rabbit α-HA mAb C29F4.

### Quantification of SCAM results

To determine the relative amount of NS4B protein modified by PEG-maleimide upon cell permeabilization with digitonin in SCAM, signal intensities in the Western blots were measured using ImageJ software (NIH, Bethesda, MD, USA). The ratio of modified NS4B (NS4B*) to non-modified NS4B was calculated and normalized to the value of aa R345C, considered to be cytoplasmic. For the differentiation of amino acid positions accessible or non-accessible for modification, arbitrary cut-off limits for the NS4B*/NS4B ratio were set at 15% of the NS4B*/NS4B signal intensity of R345C for non-accessible positions and at 30% for accessible positions. Amino acid positions with ratios in between these limits were defined as partially accessible.

### N-linked glycosylation assay

For N-linked glycosylation of NS4B, expression constructs were designed by insertion of the EC4 loop sequence containing the motif “NSS” as described above. After protein expression of transfected MVA-T7^pol^-infected Huh7-T7 cells for 24 h, cells were harvested and lysed in 50 µL of cold lysis buffer as described above, and the supernatant was divided equally. Afterwards, one-half of each sample was treated with peptide-*N*-glycosidase (PNGase F, NEB, Frankfurt/Main, Germany) according to the manufacturer’s protocol with the following optimizations. Proteins were denatured in 1× glycoprotein denaturing buffer (0.5% SDS, 40 mM DTT) by incubation for 10 min at 37°C. The lysates were treated with 250 units PNGase F in 1× GlycoBuffer 2 (50 mM sodium phosphate, pH 7.5) with 1% NP-40 added for 2 h at 37°C. Then, both sample portions were mixed with SDS sample buffer to a total volume of 80 µL, and 20 µL each was separated by SDS-PAGE with 10% polyacrylamide-tricine gels (70). The proteins were transferred to a nitrocellulose membrane and detected using the mouse α-HA mAb 6E2. In case of NS3-5B polyprotein expression, NS3 and NS5A were detected additionally using the mouse α-NS3/NS2-3 mAb 8.12.7 and mouse α-NS5A mAb GLBVD5A1.

### Split-GFP assay

HEK293T cells were transfected with 8 µg of the bicistronic pWPI expression plasmids described earlier and fixated after 24 h with 2% (wt/vol) paraformaldehyde (Thermo Fisher, Schwerte, Germany). The cells were permeabilized, blocked, and nuclei stained with DAPI the same way as in preparation for immunofluorescence staining. Subsequently, fluorescence at approximately 570 nm wavelength was detected with the Zeiss Axio Observer Z1 fluorescence microscope (Zeiss, Goettingen, Germany), captured with the acA5472-5gm GigE camera (Basler, Ahrensburg, Germany), and images were processed using ImageJ software (NIH, Bethesda, MD, USA).

## RESULTS

### Generation of BVDV-1 strain NCP7 NS4B proteins with epitope tag insertions

**Fig 1:**
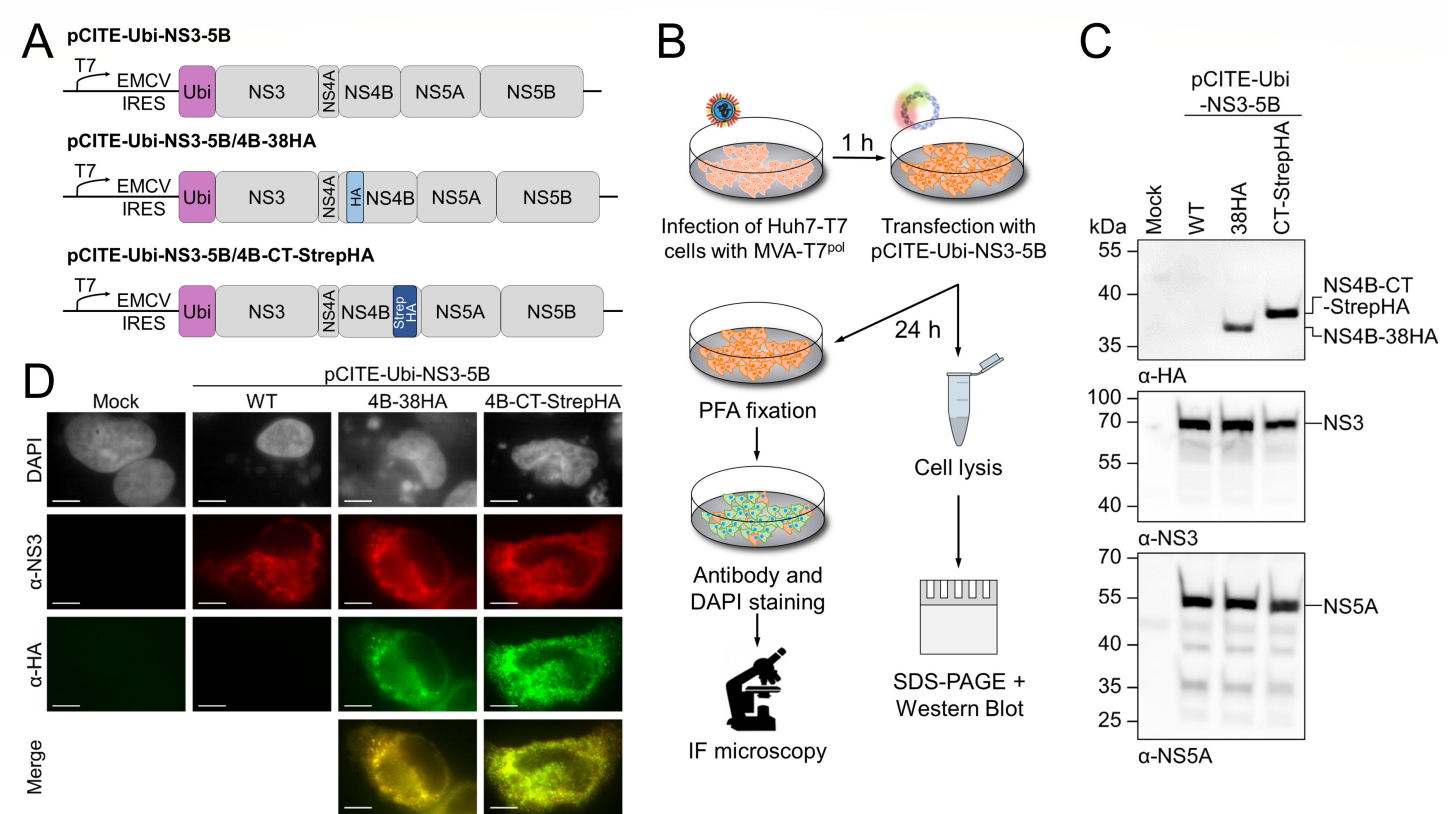
Epitope-tagged BVDV-1 NS4B variants show authentic processing of the minimal replicase polyprotein and localize similar to NS3. (**A**) Schematic representation of the BVDV-1 NS3-5B polyprotein (gray) expression constructs with insertion of an HA tag (light blue) after amino acid 38 (pCITE-Ubi-NS3-5B/4B-38HA) or a StrepHA tag (dark blue) at the C terminus with duplication of the last five NS4B amino acids (pCITE-Ubi-NS3-5B/4B-CT-StrepHA), respectively. Ubiquitin (purple) cleavage generates the authentic NS3 N terminus. (**B**) Experimental workflow. After infection with MVA encoding for the T7 RNA polymerase (MVA-T7^pol^), Huh7-T7 cells were transfected with the NS3-5B polyprotein expression constructs and harvested 24 h post-transfection (p.t.). Cells were fixed with paraformaldehyde (PFA) for immunofluorescence staining or lysed for SDS-PAGE and Western blotting, respectively. (**C**) Western blot analysis of polyprotein processing using antibodies specific for HA (top), NS3 (mid), and NS5A (bottom). Reference protein masses (kDa) are indicated on the left, and detected signals are described on the right. (**D**) Analysis of cellular localization of NS3 and the epitope-tagged NS4B variants using immunofluorescence imaging of Huh7-T7 cells 24 h p.t. at 63× magnitude. The scale bar equals 10 µm. Gray: DAPI staining of DNA; red: α-NS3/Cy3; green: α-HA/Alexa488. Merge: overlay of NS3 and NS4B staining indicated in yellow. Mock: transfection without DNA; WT: BVDV-1 NS3-5B polyprotein.

The analysis of NS4B was hampered by the absence of a mAb specific for BVDV NS4B (72). Hence, we generated modified NS4B proteins with an insertion of the HA-epitope tag at different positions to enable α-HA-specific NS4B protein detection in downstream applications (Fig. 1A).

The HA tag was introduced either after amino acid 38 (NS4B-38HA) or as the double-tag StrepHA at the C terminus of NS4B (NS4B-CT-StrepHA). In the case of NS4B-CT-StrepHA, the last 5 aa of NS4B were duplicated to ensure polyprotein processing by the NS3/4A protease.

### Polyprotein processing and localization of epitope-tagged NS4B variants

Next, we examined if NS3-mediated polyprotein processing is impaired by the modifications in NS4B in a replication-independent vaccinia virus T7 RNA polymerase (T7^pol^) expression system (Fig. 1B). The modified NS4B encoding sequences were introduced into a subgenomic cDNA encoding for the NS3-NS5B polyprotein of BVDV-1 strain NCP7 in the context of the pCITE-2a expression vector (63) (Fig. 1A). For transient expression, Huh7-T7 cells were infected by the vaccinia virus MVA-T7^pol^ (62) prior to transfection with the pCITE-Ubi-NS3-5B constructs (Fig. 1B). Polyprotein processing was evaluated by Western blot analysis using antibodies specific for NS3, NS5A, and the HA tag. As a reference for authentic polyprotein processing, a corresponding expression construct encoding wild-type (WT) NS4B was used. Cells transfected without DNA served as a control for antibody specificity. For both NS4B variants, cleaved NS3 and NS5A were detected as compared to the wild-type control, indicating proper polyprotein processing. In addition, NS4B-38HA and NS4B-CT-StrepHA showed similar expression levels in the Western blot using α-HA antibody (Fig. 1C). The localization of the epitope-tagged NS4B proteins was investigated after the expression of the NS3-5B polyprotein region using the same expression system. NS3 was used as a marker for the localization of the minimal replicase complex NS3-NS5B. Along this line, Huh7-T7 cells were fixed 24 h post-transfection, subsequently stained using NS3- and HA-specific antibodies with visualization of the nuclear DNA using DAPI. The cellular distribution of NS3 in cells expressing NS3-NS5B wild type was used as a reference. Cells transfected without DNA served as a control for antibody specificity. For the wild-type polyprotein, NS3 exhibited ER-like localization as described previously (3, 23). Both NS4B-38HA and NS4B-CT-StrepHA showed a cellular dissemination comparable to the one of NS3 (Fig. 1D).

### Effect of the epitope-tagged NS4B proteins on RNA replication and virion morphogenesis of BVDV-1

**Fig 2:**
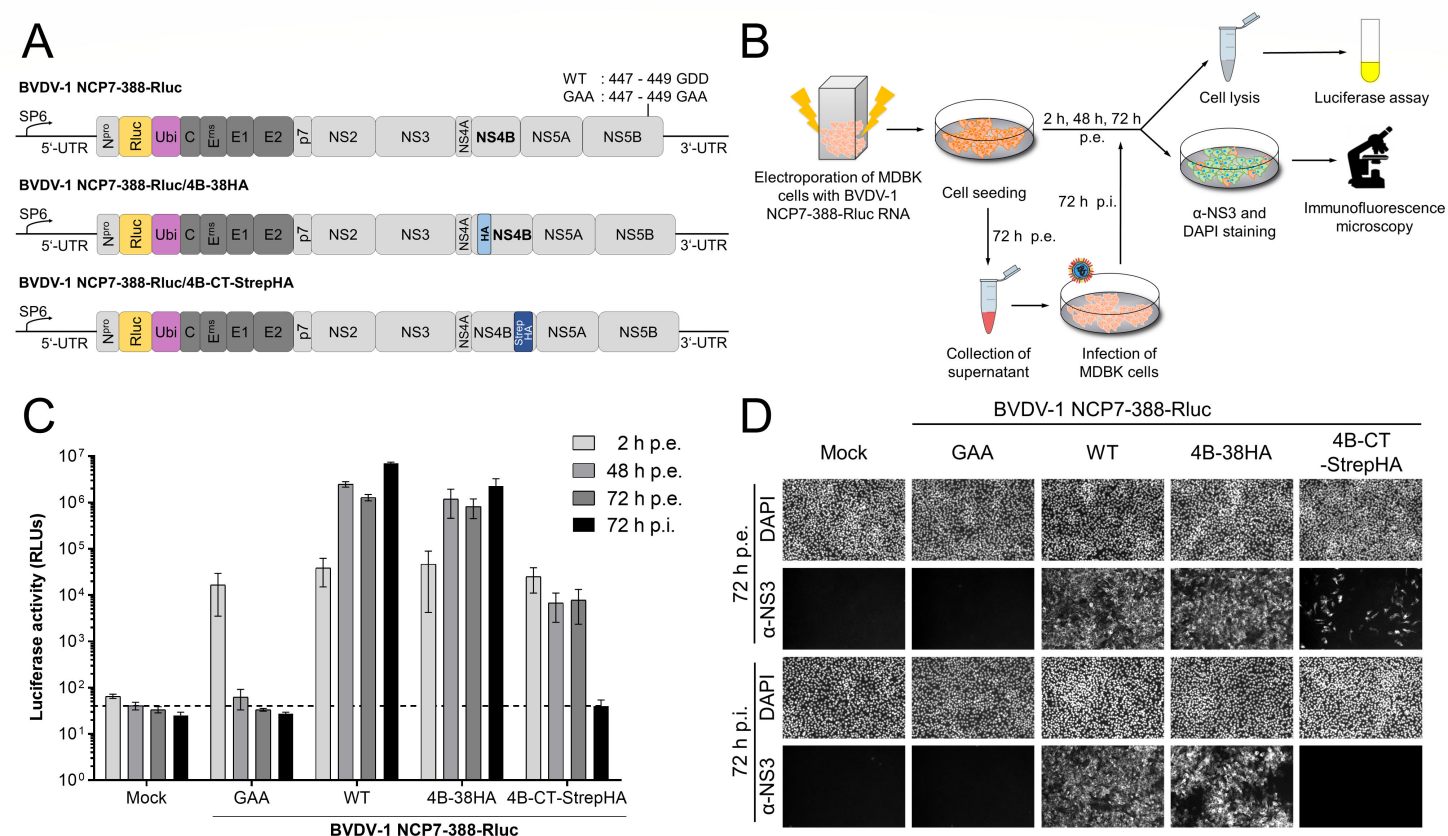
NS4B-38HA supports the entire pestiviral life cycle, while NS4B-CT-StrepHA is attenuated in RNA replication and defective in virion morphogenesis. (**A**) Genomic organization. The structural proteins (core, E^rns^, E1, E2) are indicated in dark gray, *Renilla* luciferase (Rluc) in yellow, ubiquitin (ubi) in purple, and N^pro^ as well as the non-structural proteins (p7, NS2-5B) in light gray. The encoding region is flanked by 5´ and 3´ untranslated regions (UTR), respectively. Epitope tags are indicated in blue. (**B**) Experimental workflow. MDBK cells were electroporated with *in vitro* transcribed RNA, and cells were harvested at indicated time points p.e. (2 h, 48 h, and 72 h). The supernatant 72 h p.e. was used for infection of naïve MDBK cells. At 72 h p.i., cells were harvested, and samples were analyzed for luciferase activity. (**C**) Luciferase reporter assay. RNA synthesis is measured in RLUs. Mock serves as background control, while WT and non-replicative (GAA) full-length clones were used as positive and negative control, respectively. Dashed line: RLU reference value for GAA mutant at 48 h p.e. (**D**) Immunofluorescence imaging of MDBK cells 72 h p.e. and p.i. with NS3-specific antibody. DNA was visualized using DAPI. GAA: replication-defective mutant; 4B/38 HA: HA-epitope tag after amino acid 38 of NS4B; 4B CT StrepHA: StrepHA tag at the C terminus of NS4B and duplication of the last five NS4B amino acids.

NS4B is an indispensable part of the pestiviral replicase (NS3-5B) formed at ER-derived membranes (9, 12, 14) and is also important for virion morphogenesis. It is postulated that NS4B provides crucial interaction surfaces for other replicase or virion morphogenesis components. We hypothesized that the functional positioning of these interaction surfaces is dependent on the correct integration of NS4B into these membranes (19). To prove that our generated epitope-tagged NS4B does not affect the functionality of the protein, including its membrane topology, we analyzed virus propagation in the full-length context carrying both HA-NS4B variants. Accordingly, we designed mutated full-length cDNA clones of BVDV-1 strain NCP7 either encoding for NS4B-38HA (BVDV-1 NCP7-388-Rluc/4B-38HA) or NS4B-CT-StrepHA (BVDV-1 NCP7-388-Rluc/4B-CT-StrepHA), respectively. In addition, the full-length cDNA clones encompass an Rluc-Ubi insertion of the luciferase reporter gene (Rluc) from *Renilla reniformis* followed by ubiquitin downstream of N^pro^ to allow a quantitative evaluation of viral RNA replication by determination of luciferase activity as well as the generation of an authentic Core N terminus by ubiquitin hydrolase cleavage between ubiquitin and Core (64) (Fig. 2A). MDBK cells were electroporated with *in vitro* transcribed RNAs from the modified BVDV-1 NCP7-388-Rluc full-length clones and harvested at defined time points p.e. (2 h, 48 h, 72 h) to analyze RNA replication (Fig. 2B). The supernatant 72 h p.e. was used to infect naïve MDBK cells. Infected cells were harvested 72 h p.i. to assess virion morphogenesis (Fig. 2B). Viral RNA replication was detected indirectly by evaluating lysates of electroporated cells in the luciferase reporter assay. NCP7-388-Rluc wild-type RNA and NCP7-388-Rluc/5B-GAA RNA, with a replication-inhibiting mutation (GAA) in the NS5B polymerase (73), were used as positive and negative controls, respectively. Cells electroporated without RNA served as control for background signal (Fig. 2C). Additionally, MDBK cells were PFA-fixed and stained using an NS3-specific antibody and DAPI 72 h p.e. to visualize RNA replication and 72 h p.i. to determine virion morphogenesis, respectively (Fig. 2D). The measured luciferase activity 2 h p.e. generated from the *in vitro* transcribed and electroporated RNAs was within the same range for all used NCP7-388-Rluc RNAs, assuring a comparable RNA input level. Both NCP7-388-Rluc NS4B RNAs exhibit viral RNA replication; however, they reached different levels compared to the NCP7-388-Rluc wild type after 48 h and 72 h p.e. (Fig. 2C). While NCP7-388-Rluc/NS4B-38HA replicated comparable to NCP7-388-Rluc wild type, the NCP7-388-Rluc/NS4B-CT-StrepHA RNA replicated approximately 100-fold less efficiently. As expected, the replication-deficient BVDV-1 NCP7-388-Rluc/5B-GAA did not replicate and showed luciferase levels comparable to mock electroporated cells at 48 h and 72 h p.e., respectively (Fig. 2C). The difference in the replication efficiency between the two NCP7-388-Rluc NS4B variants was confirmed by the immunofluorescence staining of NS3 (Fig. 2D). Together, the observed RNA replication of both NCP7-388-Rluc NS4B variants provides solid evidence for the functionality of the tagged NS4B proteins within the viral replicase.

Regarding virion morphogenesis, the full-length clone encoding NCP7-388-Rluc/NS4B-38HA showed a similar infectivity as the NCP7-388-Rluc wild-type virus, while NCP7-388-Rluc/NS4B-CT-StrepHA did not produce infectious progeny as judged by analyzing infected cells 72 h p.i. in the luciferase reporter assay (Fig. 2C) or by NS3-specific immunofluorescence staining (Fig. 2D). In order to confirm that infectious NCP7-388-Rluc/NS4B-38HA contains the HA tag, we detected the presence of the 38 HA epitope by immunofluorescence 72 h p.i. (Fig. S1). At this point, we cannot distinguish if the C-terminal insertion of the StrepHA sequence into NS4B causes a release or packaging defect. In summary, both epitope-tagged NS4B proteins are functional in viral RNA replication, indicating their native membrane integration. Therefore, these variants represent valid tools for antibody-specific detection of NS4B in downstream applications.

### Computational prediction of secondary structure elements and putative TMDs of pestiviral NS4Bs

**Fig 3:**
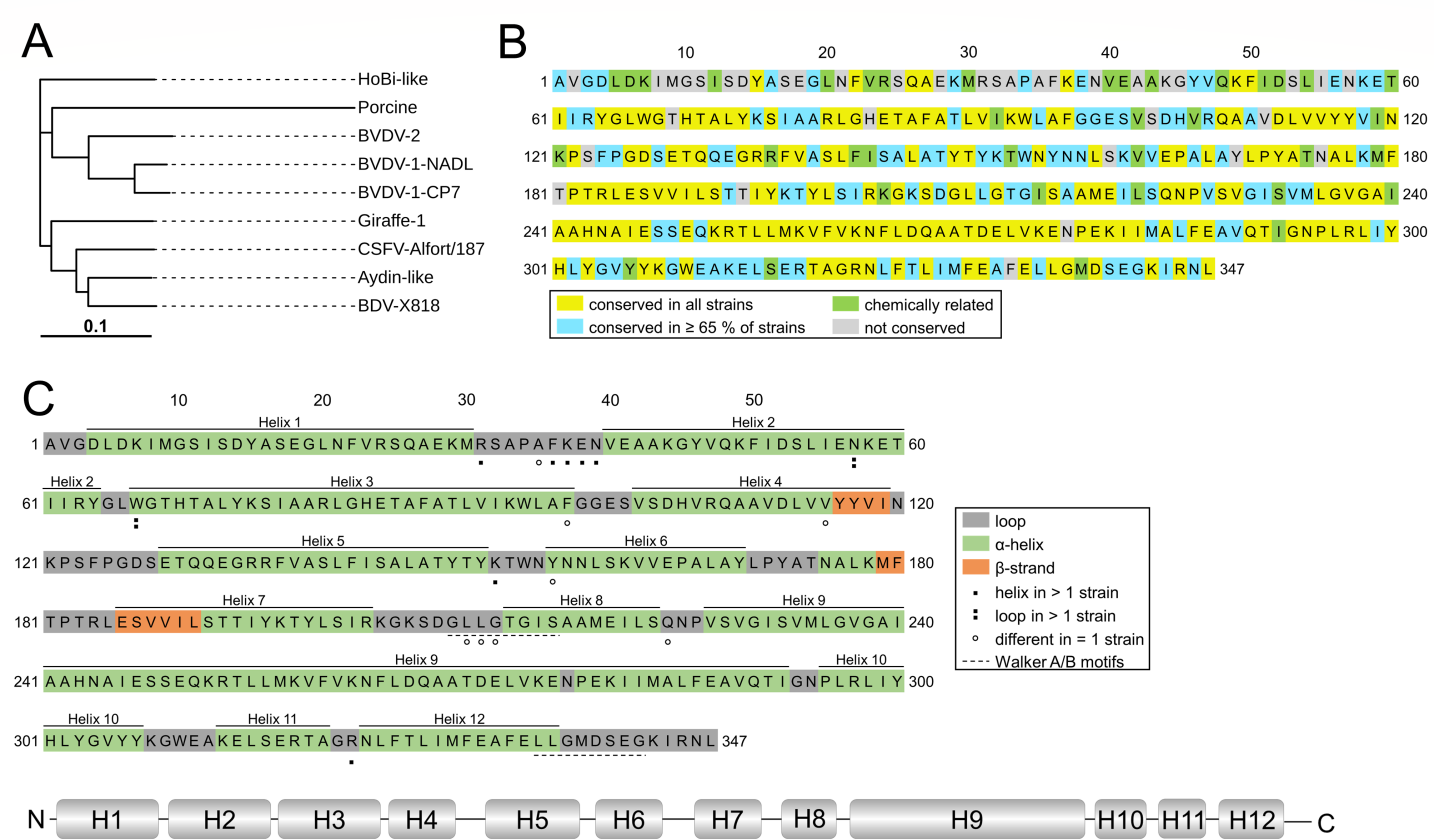
Computational prediction of secondary structure elements of pestiviral NS4Bs. (**A**) Phylogenetic tree of the pestiviral NS4B sequences used for a multiple sequence alignment (MSA) with the divergence between the genomic sequences depicted as branch length (created with iTOL). (**B**) MSA of representative pestiviral NS4B sequences performed with ClustalX2. Shown is the primary sequence of BVDV-1 strain CP7, and the degree of conservation is color-coded. (**C**) (Top) Summary of the secondary structure elements predicted with Proteus. Shown is the amino acid sequence of BVDV-1 strain CP7: α-helices (green), β-strands (orange), and loop regions (gray). Strain-specific variations are marked below. Dashed lines indicate the position of Walker A and B motifs according to Gladue et al. (27). (Bottom) Graphical summary of α-helix distribution. H: α-helix.

Prior to the experimental determination of the membrane topology of NS4B, we determined the conservation of NS4B amino acid sequences across the genus *Pestivirus*. To do so, we selected representative strains from several pestiviral species isolated from various host species (Fig. 3A) for a multiple sequence alignment of the NS4B protein sequence using ClustalX2 (41) with default parameters (Fig. 3B). The degree of conservation between the particular strains was approximately 50% with most of the conserved residues located in the C-terminal half of the protein. When the porcine pestivirus isolate “Bungowannah” was not included in the alignment, the percentage of conserved amino acids was rising to approximately 62% (Fig. S2). The highest sequence variability appeared within the first 60 amino acids of NS4B (Fig. 3B). Next, a secondary structure prediction was performed using Proteus for all the selected pestiviral strains, and the overlap was evaluated (Fig. 3C, top). The formation of secondary structure elements was comparable between the chosen strains with a high content of α-helical structures. We combined all Proteus predictions of the chosen strains (Fig. S3) into one secondary structure model for pestiviral NS4B (Fig. 3C, bottom). We used the following constraints: (i) individual helical elements should be separated by at least two amino acids; (ii) potential β-strand segments were not considered due to short length and low prediction score and incorporated into the preceding segment; (iii) α-helices shorter than 6 aa were treated as loop structures. In summary, this prediction indicates that pestiviral NS4B forms up to 12 helical elements of different lengths. Since the proposed secondary structure of NS4B was highly similar between the various species analyzed, we used the NS4B sequence of BVDV-1 strain CP7 for the prediction of potential TMDs. In total, we evaluated the output of eight different web applications in terms of the putative location of the protein’s termini and number of TMDs predicted (Table 1). Neither the localization of the N nor the C terminus was consistent between the individual tools, but a slight tendency was noticed for the N terminus to be inside and the C terminus outside of the ER lumen. The number of putative TMDs varied between 0 and 4, predominantly located toward the protein’s C terminus. Noticeably, the potential formation of a TMD in the region between aa 220 and 250 was consistent between the five applications predicting TMD formation. With respect to the NS3/4A-mediated polyprotein processing in the cytoplasm, we consider the formation of an even number of TMDs (2 or 4) with both termini on the cytosolic side to be most probable.

**TABLE 1:** Computational prediction of TMDs of pestiviral NS4Bs^*a*^

Prediction tool	Pos. of NT	Pos. of CT	No. of TMDs (aa pos.)
HMMTOP (45)	Out	Out	0
DeepTMHMM (46)	In	In	0
TOPCONS (47)	Out	Out	0
PolyPhobius (48)	In	Out	1 (**228–246**)
SPLIT (49)	–	–	1 (**227–243**)
MEMSAT-SVM (50)	In	In	2 (**224–240**, 320–336)
PredictProtein (51)	In	In	2 (**226–243**, 322–336)
MemBrain (52, 53)	Out	Out	2 (184–203, **226–254**)
	Out	Out	4 (134–146, 184–203, 208–218, **226–254**)

^*a*^ The probability of the formation of transmembrane domains was predicted for the NS4B of BVDV-1 strain CP7 using different online tools. The position of the N and C terminus across the membrane is defined as outside (out) or inside (in) of the ER lumen and the number of potentially formed TMDs is given (with the corresponding amino acid positions in parentheses). Recurrent amino acid regions are highlighted. Unavailable information is indicated as “–”.

The different results from individual transmembrane protein topology prediction tools highlight the limitations of these computational programs, at least for viral proteins, potentially due to the underrepresentation of viral sequences in the data sets used for their training. Furthermore, predictions are complicated because many TM helices in multi-spanning TM proteins are partially shielded by other TM helices. Multi-spanning TM proteins might also contain amphipathic helices that are not entirely exposed to the lipid bilayer. Accordingly, the task to generate TM topology predictions turns out to be difficult (74).

In addition, we applied AlphaFold prediction (54, 55) to pestiviral NS4B. Both available versions of AlphaFold (v.2.3.2 and v.3) yielded low confidence models for the full-length protein, with a few local arrangements of secondary structures showing slightly increased prediction confidence with regard to pLDDT value and predicted aligned error (PAE) plot (Fig. S4A and B). This is most likely due to the absence of a membrane in the prediction. To date, AlphaFold is not able to include lipid bilayers. However, both AlphaFold versions confirmed the α-helices predicted by Proteus for NS4B (Fig. S4C and D). Together, these observations highlight the need to determine the NS4B membrane topology experimentally.

### BVDV-1 NS4B forms two TMDs in the N-terminal region

**Fig 4:**
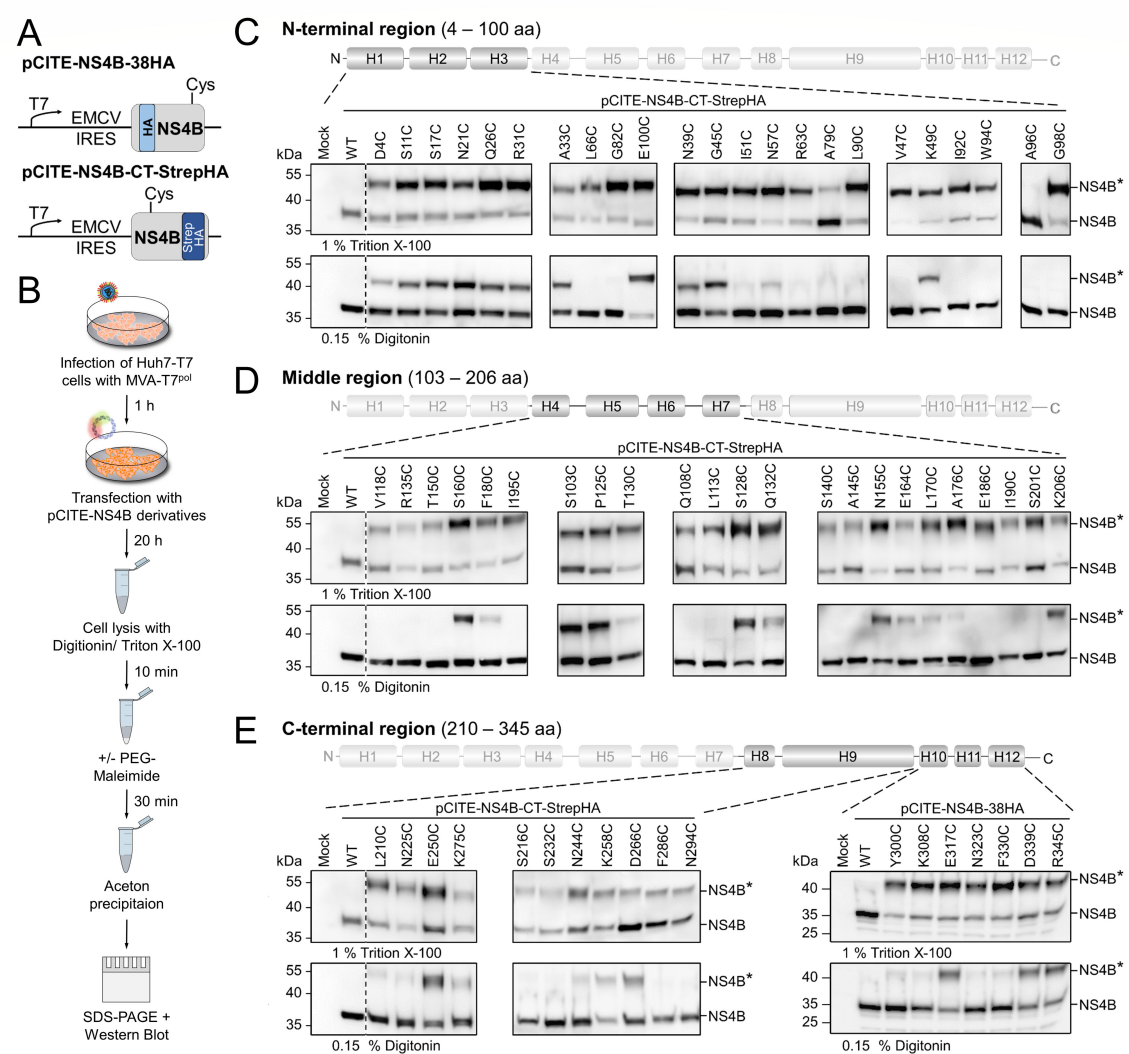
SCAM assay of the pestiviral NS4B. (**A**) Schematic overview of pCITE-NS4B-38HA and pCITE-NS4B-CT-StrepHA. A representative cysteine substitution is indicated. (**B**) Experimental workflow of the SCAM assay. MVA-T7^pol^-infected Huh7-T7 cells were transfected with the NS4B expression constructs and harvested 20 h p.t. The samples were lysed with digitonin or Triton X-100, and half of each sample was incubated with PEG-maleimide, precipitated with acetone, and analyzed by SDS-PAGE/Western blotting. (**C–E**) Western blot analysis after SCAM using an α-HA antibody of the N-terminal region (4–100 aa) (**C**), central region (103–206 aa) (**D**), and C-terminal region (210–345 aa) (**E**) of NS4B. Treatment of samples (Triton X-100 or digitonin) is indicated below each blot. Samples shown in C–E were produced in different experiments. Samples shown within one box are derived from the same blot. Mock and wild-type controls used in C–E are from one representative Western blot. The original blots for C–E, including the respective mock and wild-type samples, are provided in S7. Reference protein masses (kDa) are indicated on the left, and detected signals are described on the right. NS4B: not modified; NS4B*: PEG-maleimide modified NS4B.

To experimentally determine the membrane topology, we applied the substituted cysteine accessibility method to study the number and location of TMDs in NS4B (40). The major advantage of SCAM over other experimental tools like N-linked glycosylation assay (35) or fluorescence protease protection assay (75) is the comparably minor sequence alteration introduced into the protein of interest. SCAM requires only the exchange of an individual amino acid by a cysteine residue (in an otherwise cysteine-free protein background), whereas the other methods demand the insertion of larger peptides or the generation of fusion proteins. Wild-type BVDV NS4B does not contain cysteine residues and is therefore well suited for the SCAM assay (Fig. 3B). We generated the expression constructs pCITE-NS4B-38HA and -NS4B-CT-StrepHA and substituted single residues to cysteine by site-directed mutagenesis (Fig. 4A). The positions for exchange were evenly distributed along the protein sequence, and conserved, preferably polar or charged, amino acids were predominantly selected. Mutations upstream of NS4B amino acid position 300 were introduced into pCITE-NS4B-CT-StrepHA, whereas for downstream modifications, pCITE-NS4B-38HA was used in order to exclude an impact of the C-terminal epitope tag on the membrane topology (Fig. 4A). The transient expression of the NS4B derivatives was mediated by the MVA-T7^pol^ system described above. Therefore, Huh7-T7 cells were infected with MVA-T7^pol^ followed by DNA transfection with the expression constructs. Transfected cells were harvested 20 h post-transfection (p.t.) and equally divided into two aliquots. One aliquot was lysed with Triton X-100 to permeabilize all cellular membranes. The other aliquot was permeabilized using digitonin to allow for the selective permeabilization of the plasma membrane. Afterwards, the lysed samples were evenly divided, and only one half was treated with PEG-maleimide. Next, proteins were precipitated and assessed by Western blot analysis applying an HA-specific antibody (Fig. 4B). We confirmed the expression of the individual NS4B variants and that protein recovery after permeabilization and precipitation was comparable for both detergents (Fig. S5). The covalent modification of the introduced cysteine residue by PEG-maleimide was evaluated in the samples lysed with 1% Triton X-100 and could be detected as a size shift of the NS4B protein (NS4B*) compared to epitope-tagged NS4B (Fig. 4C through E, top panels). With the exception of mutation A96C, NS4B* was detectable for all mutants in the Triton X-100 lysed samples when treated with PEG-maleimide, demonstrating the accessibility of the introduced cysteine residues to PEG-maleimide modification. We suppose that the thiol group in the cysteine side chain at NS4B position 96 cannot be approached by PEG-maleimide because it is either buried inside of the protein or engaged in protein-protein interactions after membrane extraction. Therefore, no data for this position can be obtained by SCAM.

**Fig 5:**
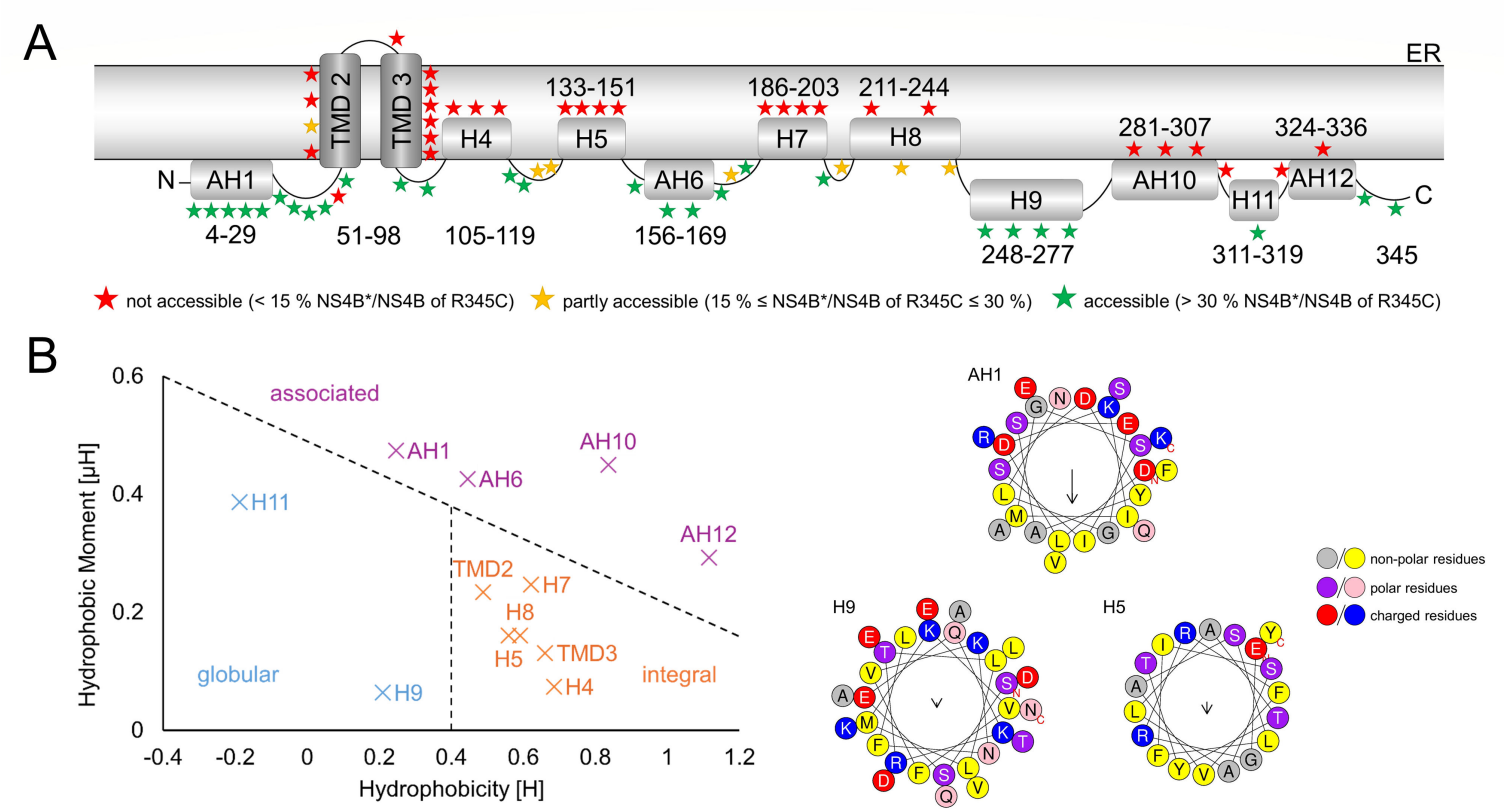
A membrane topology model of pestiviral NS4B. (**A**) SCAM-based model of the membrane topology of pestiviral NS4B with loops shown as black lines, α-helices shown in gray, and transmembrane domains highlighted in dark gray. The assumed length of helical regions is depicted. The accessibility of cysteine residues is color-coded (stars). The ratio of NS4B*/NS4B signal intensities (normalized to the value for R345C) was calculated and cut-off limits were set at 15% for inaccessibility (red) and 30% for accessibility (green). Ratios between these limits were assigned as “partly accessible” (yellow). AH: amphipathic α-helix; H: α-helix. (**B**) Adapted Eisenberg plot showing the hydrophobic moment (µH) in relation to the hydrophobicity (H) of the computational predicted, SCAM-refined α-helices of NS4B (left). The helical elements were grouped into three distinct populations according to their biochemical properties. Globular (blue): no membrane interaction; associated (purple): helices face one-sided the membrane (amphipathic); integrated (orange): helices are membrane-embedded. For each population of the Eisenberg plot, an example HeliQuest graphic (top view of the α-helix) is shown (right). Amino acids are color-coded as indicated. The arrow length depicts µH of the α-helix.

Since PEG-maleimide is membrane-impermeable, it remains in the cytoplasm. Thus, PEG-maleimide treatment after selective permeabilization of the plasma membrane in the presence of digitonin results in a selective size shift of NS4B cysteine mutants located at the cytoplasmic side of the ER membrane. To standardize the assessment of the cysteine residue accessibility, we determined NS4B*/NS4B signal intensities and normalized these values to the ratio of the R345C residue, assumed to be cytoplasmic. Residues considered inaccessible have an NS4B*/NS4B ratio below 15% compared to the R345C ratio, while accessible residues exhibit a ratio above 30% (Table S6). For mutants with cysteine substitutions close to the termini of NS4B (for the N terminus, e.g., D4C and S11C; for the C terminus, D339C and R345C), NS4B* was detected in the corresponding Western blots (Fig. 4C and E). Accordingly, the N and C terminus of NS4B are located on the cytoplasmic side of the ER membrane, which is in agreement with NS3/4A-mediated polyprotein processing in the cytoplasm. In the next step, different parts of NS4B were analyzed in a stepwise fashion using block mutagenesis (Fig. 4C through E). In the N-terminal region of NS4B (aa 33–100) we identified for two mutants, L66C and G82C, no PEG-modified NS4B (Fig. 4C). A more detailed investigation in the N-terminal region of NS4B (aa 4–100) discovered additional positions not accessible to PEG-maleimide after selective permeabilization of the plasma membrane with digitonin, namely I51C, R63C, A79C, L90C, V47C, I92C, W94C, and G98C (Fig. 4C). Together, the SCAM results showed a cohesive stretch of amino acids (NS4B aa 51–98) that are non-accessible or weakly (N57C) accessible for PEG-maleimide modification. In this region, the putative α-helices 2 and 3 are partly (helix 2) or completely (helix 3) embedded (Fig. 4C, see also Fig. 3C). We consider this section as long enough to form two transmembrane domains connected by a loop inside the ER lumen with regard to the approximated length of 20–24 aa for each membrane-spanning α-helix (76, 77). The weak accessibility of mutant N57C for PEG-maleimide modification (Fig. 4C) might indicate the orientation of its side chain toward the cytoplasm. In the middle region of NS4B, three smaller segments of consecutive mutants (Q108C, L113C, V118C; Helix H4; R135C, S140C, A145C, T150C; Helix H5; and E186C, I190C, I195C, and S201C; Helix H7) were identified as non-accessible for PEG-maleimide (Fig. 4D and 5A). Their length of around 10–15 aa was considered too short for even one transmembrane helix (Fig. 4D). The same applies to the two amino acid stretches identified within the C-terminal region of NS4B, including the mutants F286C, N294C, Y300C, K308C (AH10, Fig. 5A) as well as N323C and F330C (AH12, Fig. 5A). The segment comprising the mutants L210C, S216C, N225C, S232C, and N244C showed a pattern of alternating inaccessible to partially modifiable positions, which hinted toward a membrane association of this region (Fig. 4E). Based on the SCAM results, we developed a membrane topology that refers to the predicted 12 α-helical elements (Fig. 3C) with adjusted lengths and proposes the formation of two transmembrane domains (H2 and H3 renamed as TMD2 and TMD3, respectively) in the N-terminal region of NS4B with the N and C terminus located in the cytoplasm (Fig. 5A). Moreover, we assessed the biochemical properties of these α-helices for single NS4B proteins to distinguish between membrane-associated or membrane-embedded α-helices not crossing the membrane bilayer. To do so, the hydrophobic moment of each helical element was plotted against its hydrophobicity calculated with HeliQuest (Fig. 5B). In this adapted Eisenberg plot (78, 79), three distinct populations of α-helices were characterized: (i) “globular” defines an α-helix not interacting with the membrane; (ii) a helical element is termed “associated” if one side attaches to the outer leaflet of the membrane (amphipathic α-helix); (iii) “integral” α-helices are located inside the lipid bilayer. The calculated data points of the two transmembrane domains identified by SCAM, TMD2 and TMD3, were in the population of integral α-helices, which strengthened the conclusions drawn from the experimental data. Furthermore, 2 out of 12 putative α-helices were classified as “globular” (H9, H11), 4 as “associated,” hence described as amphipathic α-helices in the model (AH1, AH6; AH10; AH12), and 4 more α-helices as “integral” (H4, H5, H7, H8) (Fig. 5A and B). Interestingly, these integral α-helices adjoin each other pairwise in the model, which might indicate a specific role of this protein region for the function of NS4B (Fig. 5A). Whether or not NS4B dimerization/multimerization contributes to the protein’s functionality remains to be investigated.

### Dual topology of the N terminus of NS4B

**Fig 6:**
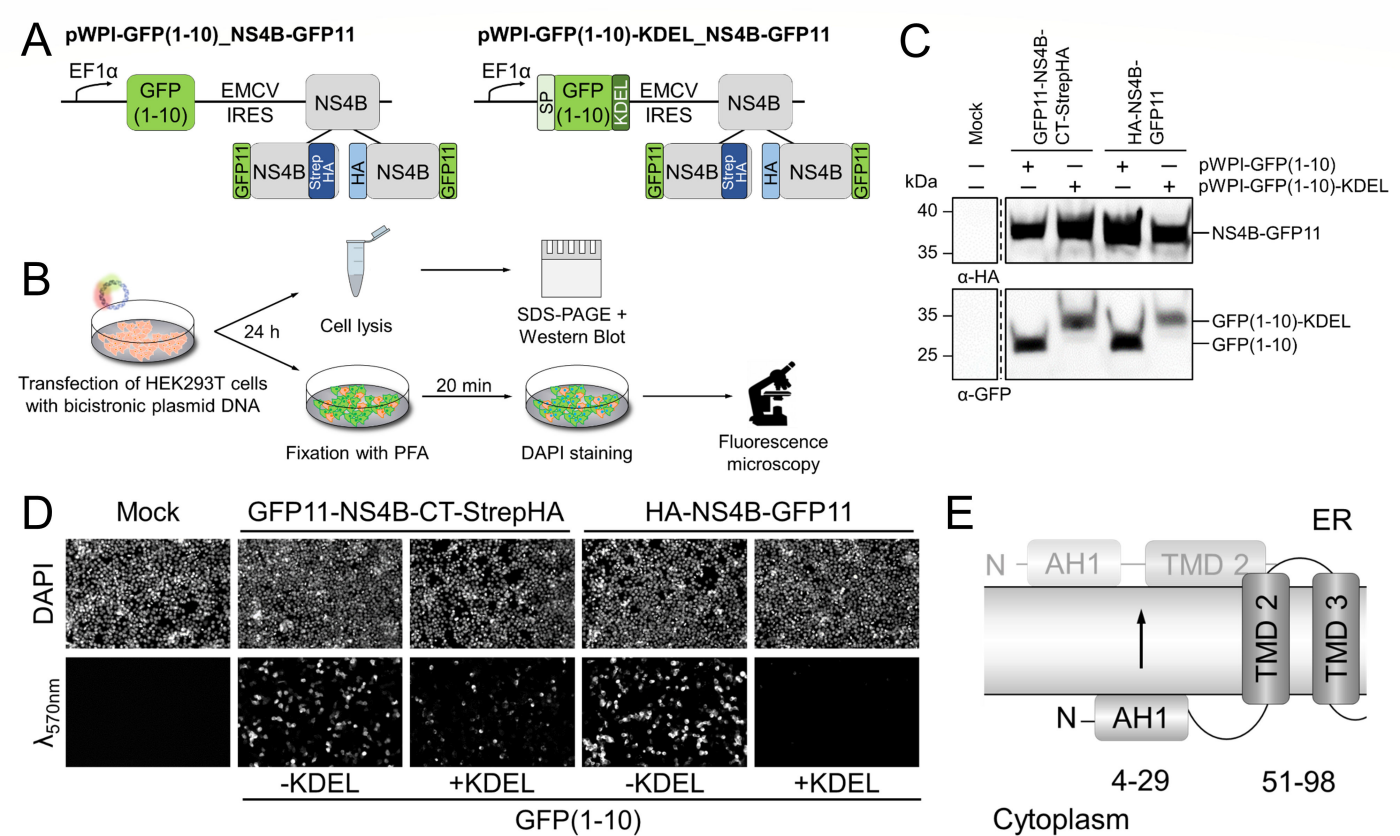
Split-GFP assay revealed dual localization of the pestiviral NS4B N terminus. (**A**) Schematic overview of the bicistronic Split-GFP expression constructs. (Left) pWPI-GFP(1-10)_NS4B-GFP11 encodes the detector molecule (GFP(1-10), green) in the first and the indicated NS4B-GFP11 fusion protein variants in the second ORF. (Right) pWPI-GFP(1-10)-KDEL_NS4B-GFP11 encodes the detector molecule with ER localization signal (GFP(1-10)-KDEL, green) in the first and the indicated NS4B-GFP11 fusion protein variants in the second ORF. The ER signal peptide (SP) and retention signal KDEL are shown in light and dark green, respectively, and NS4B epitope-tags in blue. (**B**) Experimental workflow of Split-GFP assay. HEK293T cells were transfected with the bicistronic expression constructs and fixed 24 h p.t. for DNA staining using DAPI followed by fluorescence detection. (**C**) Western blot analysis of the protein expression from the GFP(1-10)_NS4B-GFP11 constructs using an α-HA antibody (upper panel) or an α-GFP antibody (lower panel). Protein masses (kDa) are indicated on the left, and detected signals are described on the right. (**D**) Fluorescence imaging of Split-GFP analysis of the termini of BVDV-1 NS4B. GFP11-NS4B-CT-StrepHA: N-terminal NS4B-GFP11 fusion protein with C-terminal StrepHA tag insertion; HA-NS4B-GFP11: C-terminal NS4B-GFP11 fusion protein with N-terminal HA tag insertion; −KDEL: cytosolic localization of GFP(1-10); +KDEL: luminal localization of GFP(1-10). (**E**) Graphical summary of the dual localization of the N terminus of BVDV-1 NS4B (compare with Fig. 5A).

For the hepaciviral NS4B, it was shown that an N-terminal segment, including the amphipathic helix AH1 of the protein, can translocate into the ER lumen most likely by a post-translational process indicating a dual topology of HCV NS4B (34, 38). The SCAM results for the proposed AH1 of pestiviral NS4B imply the localization of the N terminus in the cytoplasm. However, ER membrane-embedded or luminal positions of substituted cysteine residues were so far only indirectly deduced by the absence of a PEG-maleimide modification of NS4B at these positions. Considering this limitation of the assay, SCAM is not appropriate to determine if such a translocation also occurs for pestiviral NS4B as observed with hepaciviral NS4B. Therefore, we further investigated the ER localization of the NS4B termini in more detail by using the Split-GFP assay (67, 68). This method utilizes the dissection of the GFP β-barrel into 10 consecutive (GFP1-10, detector) and 1 single β-strand (GFP11, sensor) and the ability of these molecules to reassociate and recreate the fluorescent protein. For this Split-GFP reassociation assay, the GFP11 sensor segment needs to be attached to the respective protein terminus to be analyzed. Co-expression of the GFP11 fusion protein with either cytoplasmic (GFP1-10) or ER-luminal-restricted [GFP(1-10)-KDEL] expression of the detector molecule allows the elucidation of the NS4B termini orientation by detecting the reconstitution of the full-length GFP by light emission. To achieve the desired co-expression, we generated a pair of bicistronic plasmids encoding in the first ORF either the detector expressed in the cytoplasm [GFP(1-10)] or in the ER lumen [GFP(1-10)-KDEL]. In the second ORF, we placed two different GFP11-NS4B fusion protein variants: either the sensor (GFP11) fused with a GS linker to the N terminus of NS4B-CT-StrepHA (GFP11-NS4B-CT-StrepHA) or to the C terminus of HA-NS4B (HA-NS4B-GFP11), respectively (Fig. 6A). To achieve high protein expression levels, HEK293T cells were transfected with the bicistronic plasmids. Twenty-four hours p.t., the cells were either harvested and analyzed by Western blotting or PFA-fixed for detection of GFP fluorescence by microscopy (Fig. 6B). Western blot analysis with antibodies specific for the HA epitope and GFP, respectively, indicated the presence of the encoded proteins from the individual ORFs in all samples in comparable amounts (Fig. 6C). Fluorescence imaging of the co-expression of GFP(1-10) with HA-NS4B-GFP11 allowed the detection of light emission, whereas upon co-expression of the luminal detector GFP(1-10)-KDEL, fluorescence was absent (Fig. 6D, right). These results are in line with a cytoplasmic localization of the NS4B C terminus and support our SCAM-based model. Moreover, the evaluation of the co-expression of GFP11-NS4B-CT-StrepHA with both detector molecules revealed the presence of fluorescence in either case, and thus the cytoplasmic and luminal reconstitution of full-length GFP by GFP11-NS4B-CT-StrepHA sensor and GFP(1-10) or GFP(1-10)-KDEL detector reassociation. The interaction between the GFP(1-10)-KDEL detector and GFP11-NS4B-CT-StrepHA sensor molecule in the ER lumen appeared to generate a weaker signal compared to the reassociation of cytoplasmic GFP(1-10) detector with GFP11-NS4B-CT-StrepHA sensor. Whether or not this observation suggests that the N-terminal translocation of NS4B into the ER occurs only to a certain degree, and if this translocation is reversible, remains to be determined (Fig. 6D, left). Of note, this dual topology is similar to the hepaciviral NS4B N-terminal topology (38). The Split-GFP data were incorporated into our membrane topology model of pestiviral NS4B with the dual positioning of AH1 and its consequence for TMD2 now shown (Fig. 6E).

### Evaluation of the NS4B membrane topology model

**Fig 7:**
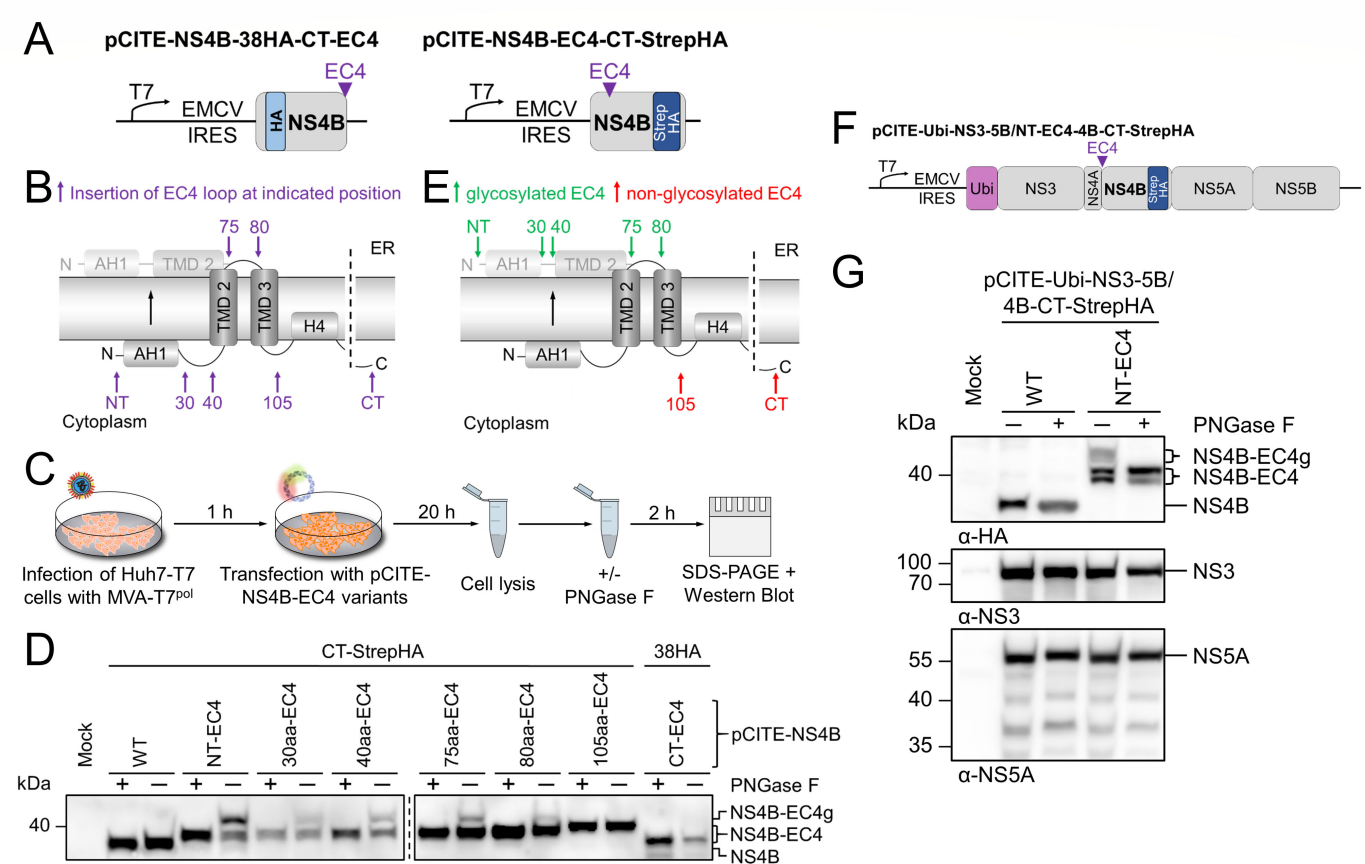
N-linked glycosylation assay validated the NS4B membrane topology model and indicated the translocation of the NS4B N terminus to occur post-translationally. (**A**) Schematic overview of the epitope-tagged (blue) pCITE-NS4B expression constructs with insertion of a glycosylation motif (EC4 loop, purple). (**B**) Graphical representation of the EC4 insertion sites (purple arrows) in the NS4B membrane topology model. (**C**) Experimental workflow of N-linked glycosylation assay. Huh7-T7 cells were infected with MVA-T7^pol^ and transfected with the expression constructs of the NS4B-EC4 variants. Samples were lysed 20 h p.t., and half of each sample was treated with PNGaseF prior to SDS-PAGE and Western blotting. (**D**) Western blot analysis of N-linked glycosylation assay of NS4B-EC4 mutants using an α-HA antibody. Reference protein masses (kDa) are indicated on the left, and detected signals are described on the right. (**E**) Visualization of the EC4 loop glycosylation in NS4B as assessed by Western blotting (compared to D). Green: EC4 loop is glycosylated while present in the ER lumen; red: EC4 loop is non-glycosylated due to the presence in the cytoplasm. (**F**) Schematic representation of the BVDV-1 NS3-5B polyprotein encoding NS4B-CT-StrepHA (blue) with insertion of an N-terminal EC4 loop with upstream repeat of the first 5 aa (pCITE-Ubi-NS3-5B/NT-EC4-4B-CT-StrepHA). (**G**) Western blot analysis of N-linked glycosylation assay in the context of the NS3-5B polyprotein using antibodies specific for HA (top), NS3 (mid), and NS5A (bottom). Reference protein masses (kDa) are indicated on the left, and detected signals are described on the right. NS4B-EC4: non-glycosylated NS4B-EC4 fusion protein; NS4B-EC4g: glycosylated NS4B-EC4 fusion protein.

So far, the absence of PEG-maleimide modification was used as indication for a location of the residue in the ER lumen or in close association to the ER membrane. To obtain a selective modification of positions localized in the ER lumen, the N-linked glycosylation assay, previously also used to establish the membrane topology model for hepaciviral NS4B, was applied (35). This assay is based on the insertion of an established glycosylation acceptor site (including the essential motif N-X-S/T), such as the 30-residue-long peptide sequence of the EC4 of the human erythrocyte anion exchanger 1, into the protein of interest, such as NS4B (Fig. 7A) (65). This relatively large insertion generates a minimal distance of 12 to 14 amino acids from the membrane to the acceptor Asn necessary for efficient glycosylation to occur (80). Due to their size, such insertions can affect the secondary structure and consequently alter the membrane topology of a protein if placed randomly. Based on our topology model, we selected positions for the EC4 loop insertions within the N-terminal NS4B region (Fig. 7B). This approach aimed at providing further evidence for the translocation of the N terminus of NS4B into the ER lumen as well as for the formation of the postulated transmembrane domains TMD2 and TMD3. Accordingly, the sequence encoding for the EC4 loop was inserted at selected positions into the pCITE-NS4B-CT-StrepHA expression vector. Additionally, the EC4 loop, seamlessly fused to either end of NS4B, was expressed by pCITE-EC4-NS4B-CT-StrepHA and pCITE-NS4B-38HA-CT-EC4, respectively (Fig. 7A). The modified NS4B variants were transiently expressed utilizing the MVA-T7^pol^ system (Fig. 7C). Huh7-T7 cells were infected with MVA-T7^pol^ 1 h prior to transfection with the generated NS4B-EC4 expression constructs. The cells were harvested 20 h p.t. and evenly divided for the treatment of one-half with PNGase F to remove N-linked glycans. The samples were assessed by Western blot analysis with an antibody specific for the HA epitope (Fig. 7C). In the case of N-glycosylation, the expected gain in molecular weight of NS4B was approximately 2 kDa if the addition of the smallest glycan moiety (Man_9_GlcNAc_2_) in the ER lumen is assumed (81). Cells transfected with pCITE-NS4B-CT-StrepHA without EC4 insertion (labeled WT) served as control (Fig. 7D). The results obtained for the N-terminal and the C-terminal NS4B-EC4 mutants, respectively, corroborated the developed NS4B membrane topology model: for NS4B-NT-EC4-CT-StrepHA, a gain in molecular mass as a signal for N-glycosylation of NS4B (NS4Bg) was detected; this additional molecular mass was lost after PNGase F treatment. In contrast, no indication of N-glycosylation was found for the C-terminal fusion of EC4 to NS4B (NS4B-38HA-CT-EC4, Fig. 7D), further supporting a cytoplasmic localization of the NS4B C terminus. To evaluate if the luminal localization of the N terminus occurs post-translationally, we determined glycosylation in the context of the NS3-5B/4B-NT-EC4-CT-StrepHA polyprotein (Fig. 7F). We hypothesized that N-terminal glycosylation can only happen after NS3/4A-mediated polyprotein processing in the cytoplasm. To retain an intact NS3/4A cleavage site between NS4A and NS4B, the first 5 aa of NS4B were repeated upstream of the N-terminal EC4. After expression and PNGase F treatment, we detected proper polyprotein processing of NS3-5B/4B-NT-EC4-CT-StrepHA (Fig. 7G, mid/bottom panel) and EC4-specific glycosylation (Fig. 7G, top panel), thus confirming a dual topology of the NS4B N-terminal region. Of note, we observed a double band for the glycosylated and the non-glycosylated NS4B-EC4 (Fig. 7G, top panel). This is most likely due to an alternative NS3/4A cleavage site within the EC4 sequence. For all samples of NS4B mutants with an internal EC4 loop insertion (30 aa-EC4, 40 aa-EC4, 75 aa-EC4, 80 aa-EC4), with the exception of variant 105 aa-EC4, an additional signal corresponding to NS4Bg was distinguished in WB (Fig. 7D). The glycosylation of the 30 aa- and 40 aa-EC4 loop NS4B variants was in line with the expected shift of the TMD2 sequence from membrane-integral to putatively membrane-associated at the luminal side of the bilayer upon translocation of the NS4B N terminus (Fig. 7E). The observed glycosylation of the 75 aa- and 80 aa-EC4 loop NS4B variants is in line with the presence of a luminal loop structure connecting TMD2 and TMD3 (Fig. 7E). However, the fraction of glycosylated vs non-glycosylated NS4B signal intensities for these mutants is prominently weaker compared to other variants (e.g., NS4B-40aa-EC4). A possible explanation is that also in the context of the EC4 loop, N-glycosylation efficiency is considerably influenced by structural constraints like the distance to the ER membrane at the respective NS4B position (80). The fact that the NS4B-105aa-EC4 mutant was not found to be N-glycosylated further strengthens the proposed position of the transmembrane domains in the model (Fig. 7D and E).

## DISCUSSION

For the pesti- and hepaciviruses, viral RNA synthesis requires the formation of the minimal replicase complex composed of NS3-5B as well as cellular co-factors at restructured ER membranes (22, 82). In the life cycle of HCV, NS4B is directly involved in membrane remodeling (15) and fulfills crucial functions in genome replication and virion morphogenesis (9, 12). For pestiviruses, the mechanisms by which NS4B supports viral RNA synthesis and virion morphogenesis are less investigated. However, as a multi-spanning integral membrane protein (19), the orientation of NS4B within intracellular membranes is critical to establish functional viral-viral as well as viral-cellular protein-protein interactions during the different stages of the viral life cycle. Here, we present an experimental membrane topology model for pestiviral NS4B, in which the N terminus and C terminus reside in the cytoplasm and two predicted transmembrane domains (TMD2–3) are formed in the N-terminal region of the protein followed by nine putative α-helices with different degrees of membrane association (Fig. 5A). Moreover, we demonstrated a dual topology of the N-terminal amphipathic α-helix (AH1) and proposed a reorientation of TMD2 as a consequence of AH1 translocation (Fig. 6E).

As pre-requisite for studying NS4B membrane topology, we established two HA-tagged protein variants, NS4B-38HA and NS4B-CT-StrepHA, to allow antibody-specific protein detection (Fig. 1 and 2). While the full-length clone BVDV-1 NCP7-388-Rluc/4B-38HA showed a comparable replication efficiency and virion morphogenesis to wild-type virus, the NCP7-388-Rluc/4B-CT-StrepHA variant was considerably impaired in viral RNA synthesis and lacked detectable production of infectious viral particles (Fig. 2C and D). Due to the low RNA replication capacity of NCP7-388-Rluc/4B-CT-StrepHA, it remains unclear whether or not the C-terminal StrepHA insertion also causes a specific defect in genome packaging/virion assembly or particle release. In HCV, critical self-interactions mediated by the C-terminal region of NS4B (16, 20) and protein-protein interactions with the N-terminal region of NS5A were described to impact RNA replication (30). Therefore, the observed negative effect of the C-terminal NS4B HA tag on pestiviral RNA replication may indicate the functional importance of similar contacts formed during genome replication, despite intact polyprotein processing (Fig. 1C). Nevertheless, both variants support viral RNA replication and are thus suitable for their application in defining the membrane topology of NS4B.

The comparison of our experimental SCAM assay-based membrane topology model for pestiviral NS4B displays similarities and notable differences to the current NS4B membrane topology model of the closely related HCV. For both proteins, an array of helical secondary structure elements has been predicted that are either membrane-associated or -embedded (Fig. 5A) (29, 35, 38). Importantly, our analysis not only confirmed the orientation of the N and C terminus toward the cytoplasm but also demonstrated that AH1 in BVDV NS4B can undergo a post-translational translocation to the ER lumen similar to the first α-helix AH1 in HCV NS4B (Fig. 5A and 7G) (29, 35, 38). Besides these similarities, HCV and BVDV NS4B differ in three main aspects: (i) in the pestiviral NS4B model, two transmembrane domains are postulated to form in the N-terminal part, while four TMDs are predicted to form in the center of HCV NS4B; (ii) the two putative TMDs are followed by a C-terminal region with nine predicted α-helical elements in the pestiviral protein compared to two (H1 and H2) in hepaciviral NS4B, which is in accordance with the size difference of the two proteins; (iii) in HCV NS4B, these non-TMD helices are either postulated to be cytoplasmic (H1) or membrane-associated (H2), while four helical elements appear to be membrane-embedded in BVDV NS4B (Fig. 5A) (35). Comparison of the membrane topologies between pestiviral NS4B and the NS4B proteins of Zika virus and DENV from the genus *Orthoflavivirus* shows almost no similarities. For these proteins, five TMDs connected by loop structures without additional secondary structure elements have been suggested. Both termini were postulated to reside in the ER lumen, and there are indications of a repositioning of the C terminus toward the cytoplasm (39, 83, 84).

The computational predictions of the pestiviral NS4B transmembrane domains were inconsistent between the various tools applied. They indicated up to four TMDs primarily in the C-terminal region of the protein (Table 1). The programs suggested aa 224 to 254 (mainly located in H8) as recurrent positions for a membrane-spanning α-helix, mainly based on the biophysical properties of H8 depicted in an Eisenberg plot (Fig. 5B). These properties were placing H8 as a membrane-integral α-helix in the same distinct population as the TMDs 2 and 3. However, the formation of such a membrane-spanning α-helix was not confirmed by our experimental SCAM data that indicated the α-helix H8 to be most likely membrane-embedded but not crossing the inner membrane leaflet (Fig. 5A). In particular, the helix length and the alternating accessibility of the analyzed amino acids in H8 to PEG-maleimide modification are contradictory to the predicted TMD formation.

In the pestiviral life cycle, the processing of the polyprotein region NS3-NS5B by the NS3/4A serine protease requires the protein junctions NS4A/4B and NS4B/5A to be located in the cytoplasm to be accessible for cleavage (85). Therefore, the N and C termini of NS4B must be present in the cytoplasm during polyprotein processing. Only the tool MemBrain (52, 53) predicted this requirement for a topology model with two or four transmembrane domains, respectively. While MEMSAT-SVM (50) and PredictProtein (51) claimed the termini to be inside the ER lumen, PolyPhobius (48) proposed a cytoplasmic location of the C terminus alone (Table 1). In contrast to these predictions, our experimental SCAM data supported the assumption of a cytoplasmic orientation of the N and C termini of NS4B (Fig. 4C and E and 5A). The Split-GFP assay strengthened these conclusions (Fig. 6D). Interestingly, this assay also indicated the N terminus of a certain fraction of NS4B to be localized in the ER lumen (Fig. 6D). The shift of AH1 from the cytoplasmic to the luminal side of the ER requires the reorientation of TMD2 from inside the membrane toward the ER-facing leaflet (Fig. 6E). However, this rearrangement could not be directly tested by SCAM since membrane-integral and luminal cysteine residues cannot be modified by PEG-maleimide after selective permeabilization and thus were only indirectly assigned by the absence of modification. Similar observations were described for HCV NS4B (29, 34, 38). In the case of HCV NS4B, a dual topology of the N terminus was shown for *in vitro* translated NS4B integrated into microsomal membranes by an N-linked glycosylation assay (38) and later also observed by immunofluorescence imaging upon selective permeabilization of a functional HCV replicon with an HA tag inserted between AH1 and AH2 (29). Our *in cellulo* application of the N-linked glycosylation assay with a NS4B variant encoding an N-terminal glycosylation motif (NS4B-NT-EC4-CT-StrepHA) also supported a dual N-terminal topology for pestiviral NS4B, while the glycosylation of the internal insertions of the EC4 peptide into the loop region connecting AH1 and TMD2 (NS4B-30aa-EC4-CT-StrepHA; NS4B-40aa-EC4-CT-StrepHA) is providing evidence for the postulated reorientation of TMD2 to the ER lumen (Fig. 7D). The functional implications of this translocation for pestiviral RNA synthesis and/or virion morphogenesis remain to be addressed. An attractive yet unproven hypothesis could be that the translocation into the ER lumen is required for NS4B functions during virion morphogenesis. Also, the question arises if this translocation is a reversible process and, if so, whether it is regulated by determinants in NS4B itself or by surrounding and/or interacting elements, respectively. Notably, there is evidence for cellular ER membrane proteins that the inversion of a protein’s membrane topology is correlated to the function of this protein (86, 87). Additionally, an influence of fatty acids like ceramide on such alternative membrane topologies was stated (87). For CSFV, Tamura et al. created a chimeric virus based on the vaccine strain GPE- with a replacement of the NS4B region by the sequence of the highly virulent strain Eystrup. Five of the seven identified aa variations between the NS4B sequences of the two strains were located in a putative α-helix (α2) spanning aa 41–80. It was shown that this chimeric virus has an elevated replication *in cellulo* and virulence *in vivo* compared to the vaccine strain (88). However, the underlying mechanism was not further investigated. These data indicate a role of the N-terminal region of NS4B for viral replication efficiency and virulence enhancement. It is tempting to speculate that this effect is related to an impact (positive or negative) on the dual topology of the NS4B N terminus (88). A potential role of this N-terminal translocation for the NS4B-induced membranous web formation in the hepaciviral life cycle was suggested (29). Interestingly, NS4B-induced rearrangement of host membranes has also been proposed to occur during pestiviral replication (19). Furthermore, homotypic oligomerization of HCV NS4B via the N-terminal AH1 (16) and residues in the linker regions between AH1 and AH2 as well as by residues within AH2 (31) has been shown to have functional implication into HCV replicase assembly (16). If a similar AH1-mediated oligomerization exists for BVDV NS4B, it has not been experimentally addressed. Of note, the potential capability of BVDV NS4B to oligomerize makes it an attractive candidate platform for viral replicase assembly. In summary, the N-terminal α-helix AH1 of pestiviral NS4B has comparable characteristics to its hepaciviral ortholog, which supports the membrane attachment of the protein due to its amphipathic property and serves as a platform for oligomerization. The individual contributions of the dual topologies of the protein in viral RNA synthesis and virion morphogenesis in both virus systems remain yet to be determined.

In contrast to the computational TMD prediction, our SCAM data indicated the formation of two transmembrane domains spanning from aa 51 to 98. However, we cannot exclude that the TMD region in pestiviral NS4B expands slightly further toward the N or C terminus, respectively. This limitation is based on our observation that aa 47 was inaccessible for modification in SCAM while aa 45 and 49 are clearly modified (Fig. 4C and 5A; Table S6). Of note, our experimental membrane topology depicts a two-dimensional model of how NS4B spans through the membrane, but there still exists a third dimension in which the protein can be tilted (89). However, the presence of complex or irregular patterns of accessibility can be more difficult to interpret. Such patterns might suggest the presence of kinks, twists, and dynamic changes in the structure of the membrane-spanning or -associated segments. Accordingly, TMD2 might also include the region from aa 47 to 50, while TMD3 could extend beyond aa 98. The data from our N-linked glycosylation assay confirmed that, for aa 75 and 80, the inserted EC4 loop is available for glycosylation, but the modification is less efficient compared to the NS4B derivatives with EC4 insertion at the N-terminal region preceding AH1 and at the aa position 30 or 40 in the loop between AH1 and TMD2, likely due to their close membrane proximity (Fig. 7D) (80). Moreover, the insertion of the EC4 peptide after aa 100, which is postulated to reside in the cytoplasmic loop following TMD3, did not lead to detectable glycosylated NS4B in the Western blot (Fig. 7D). Together, these data suggest the formation of a TMD region from aa 51 to 98 of NS4B, and this TMD formation is consistent with weak glycosylation in the luminal loop as access to the glycosylation motif is restricted in this case due to the membrane proximity of the amino acids (80). However, statements about the exact length of the individual elements (i.e., TMD2, TMD2–3 loop, TMD3) are not possible with the presented data. Refinement of these aspects requires additional structural information from advanced methods like X-ray crystallography or cryo-electron microscopy, which come both with their unique obstacles especially regarding membrane proteins (90).

As mentioned earlier, the SCAM-based membrane topology model reflects only a two-dimensional view on the orientation of pestiviral NS4B across the ER membrane. It should be considered that the protein might be involved not only in homotypic interactions like oligomerization (as reported for HCV NS4B), but also in intramolecular interactions like helix-helix bundles with consequences for the membrane topology. The unique arrangement of the consecutive membrane-integral α-helices H4/H5 and H7/H8 interspaced with the amphipathic α-helix AH6 implies such interactions that might also form platforms for specific protein-protein interactions with viral proteins (17) or non-defined cellular host factors. Similar considerations of an alternating membrane association apply for the region H9/AH10/H11/AH12, which might be in favor of the establishment of important protein-protein interactions. Of note, a recent study on DENV NS4B reported an NS5B-dependent shift of the last two predicted α-helices from membrane-embedded to transmembrane with translocation of the C terminus into the cytosol (84).

Although in pestiviruses the formation of ER membrane alterations comparable to the membranous web of HCV was not described yet (19, 23), the proposed topology of BVDV-1 NS4B in our model indicates its potential to induce the membrane curvature required for the building of the replication factories, which are prominent for many members of the family *Flaviviridae* (22). Especially, amphipathic structures similar to the amphipathic helices AH1, AH2, and H2 found at the N- and C-terminal ends of HCV NS4B (e.g., BVDV NS4B AH1, TMD2, AH10, and AH12) might be involved in membrane association. These exposed amphiphilic domains could be sufficient to induce the membrane disordering postulated for the formation of the virus replication complex. Membrane bending might be dependent also on oligomerization as seen in HCV (29, 31).

In summary, our study provides the first membrane topology model for pestiviral NS4B based on the substituted cysteine accessibility method showing remarkable differences to the closely related HCV. The protein is predominantly membrane-anchored by two transmembrane domains in the N-terminal region, and their position was further validated by the analysis of glycosylation acceptor site recognition. While the C terminus resides in the cytoplasm, Split-GFP data indicated a reorientation of the N terminus from the cytoplasm to the ER lumen, which was also confirmed by the glycosylation assay. Our findings highlighted a unique arrangement of α-helical structures toward the C terminus of NS4B that implies an involvement in diverse protein-protein interactions as well as potential membrane remodeling as seen by other members of the family *Flaviviridae*. The insights into pestiviral NS4B membrane topology presented here provide the foundation for in-depth mechanistic analyses of its essential functions in viral RNA synthesis and virion morphogenesis and its potential impact on virus persistence.

## SUPPLEMENTAL MATERIAL

Supplemental material jvi.00825-25-s0001.docx Figures S1 to S7; Table S1.

ASM does not own the copyrights to Supplemental Material that may be linked to, or accessed through, an article. The authors have granted ASM a non-exclusive, world-wide license to publish the Supplemental Material files. Please contact the corresponding author directly for reuse.
