# Supplementary material for: Deciphering the membrane topology of the pestiviral non-structural protein 4B (NS4B)
Source: J Virol. 2025 Aug 13;99(9):e00825-25. doi: 10.1128/jvi.00825-25 (PMC12455984; doi:10.1128/jvi.00825-25)
Supplement: Supplemental material — Figures S1 to S7; Table S1. [file jvi.00825-25-s0001.docx]

**
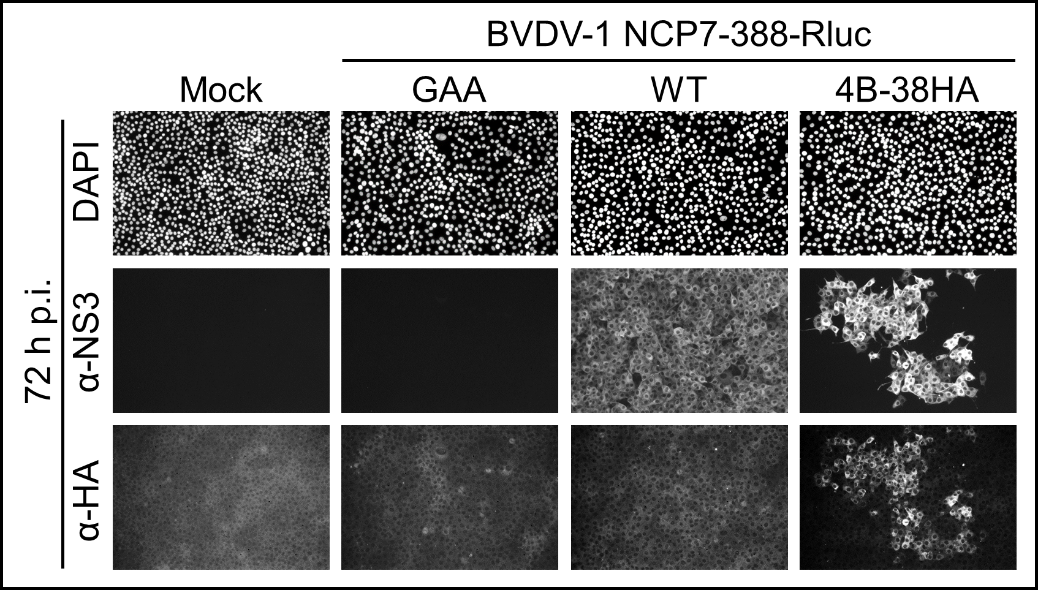
**

**Fig S1) Functional validation of the HA-epitope in the full-length clone NCP7-388-Rluc/NS4B-38HA in MDBK cells.** Immunofluorescence imaging of MDBK cells 72 h p.i. stained for NS3/Cy3 and HA/Alexa488. DNA was visualized using DAPI. WT: wild type; GAA: replication-defective mutant; 4B/38HA: HA-epitope tag after amino acid 38 of NS4B.


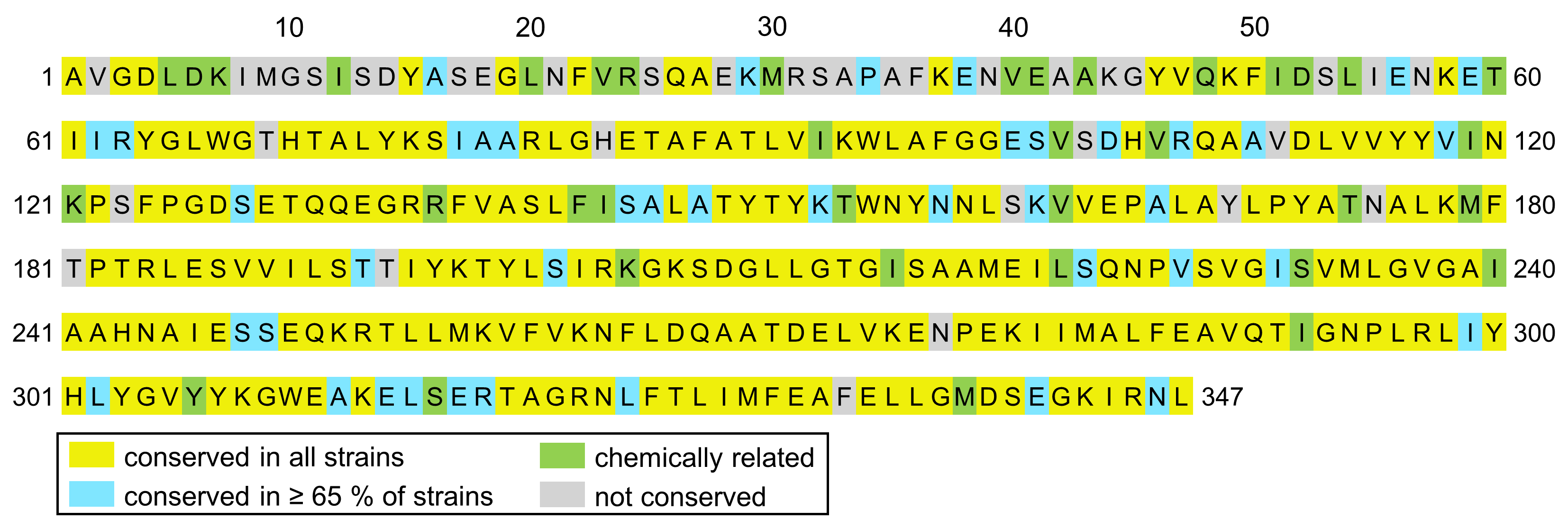


**Fig. S2) Multiple sequence alignment of representative pestiviral NS4B sequences.** Performed with ClustalX2 with exclusion of Porcine pestivirus (PPeV) Bungowannah. Shown is the primary sequence of BVDV-1 strain CP7 and the degree of conservation is color-coded.


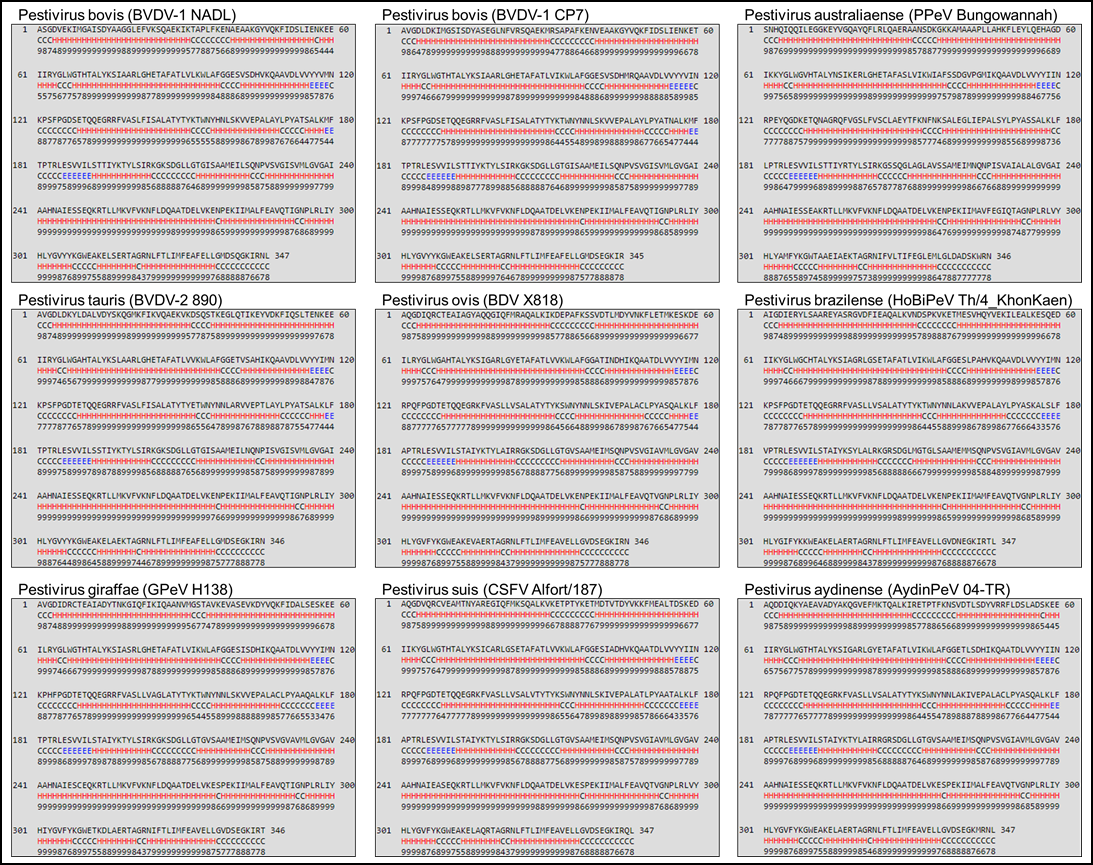


**Fig. S3) Secondary structure prediction of pestiviral NS4Bs.** Output files of the web application Proteus using the amino acid sequence (upper row) of NS4B from various pestiviral strains as input. The predicted structural element (middle row) and the probability (bottom row) are indicated. C: coiled/loop; E: β-strand; H: α-helix.

**
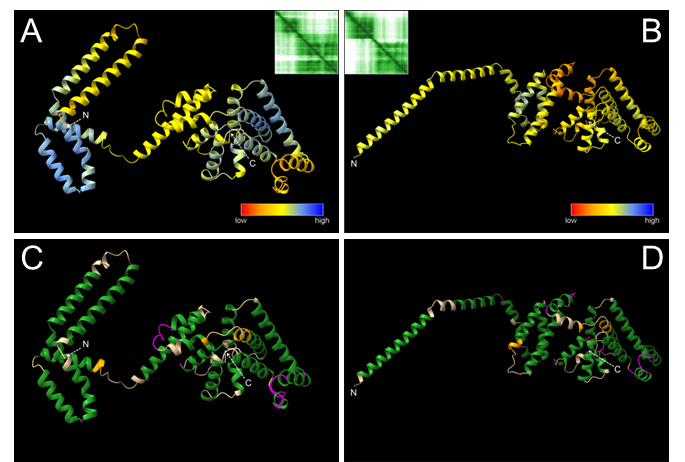
**

**Fig. S4) Alphafold prediction of BVDV-1 NS4B structure.** (A-B) Highest-ranked structure model using AlphaFold v2.3.2 (A) and v3 (B) colored by pLDDT value (local confidence) as indicated below. The corresponding PAE plots (global confidence) are depicted with the color key ranging from white to green – high to low predicted error for each amino acid. (C-D) Same structure models as in A-B showing a comparison of the secondary structure prediction between Proteus (compare Fig 3C) and AlphaFold v2.3.2 (C) or v3 (D), respectively. Green: α-helix in both predictions; Purple: α-helix only for Proteus; Orange: predicted β-strand by Proteus.


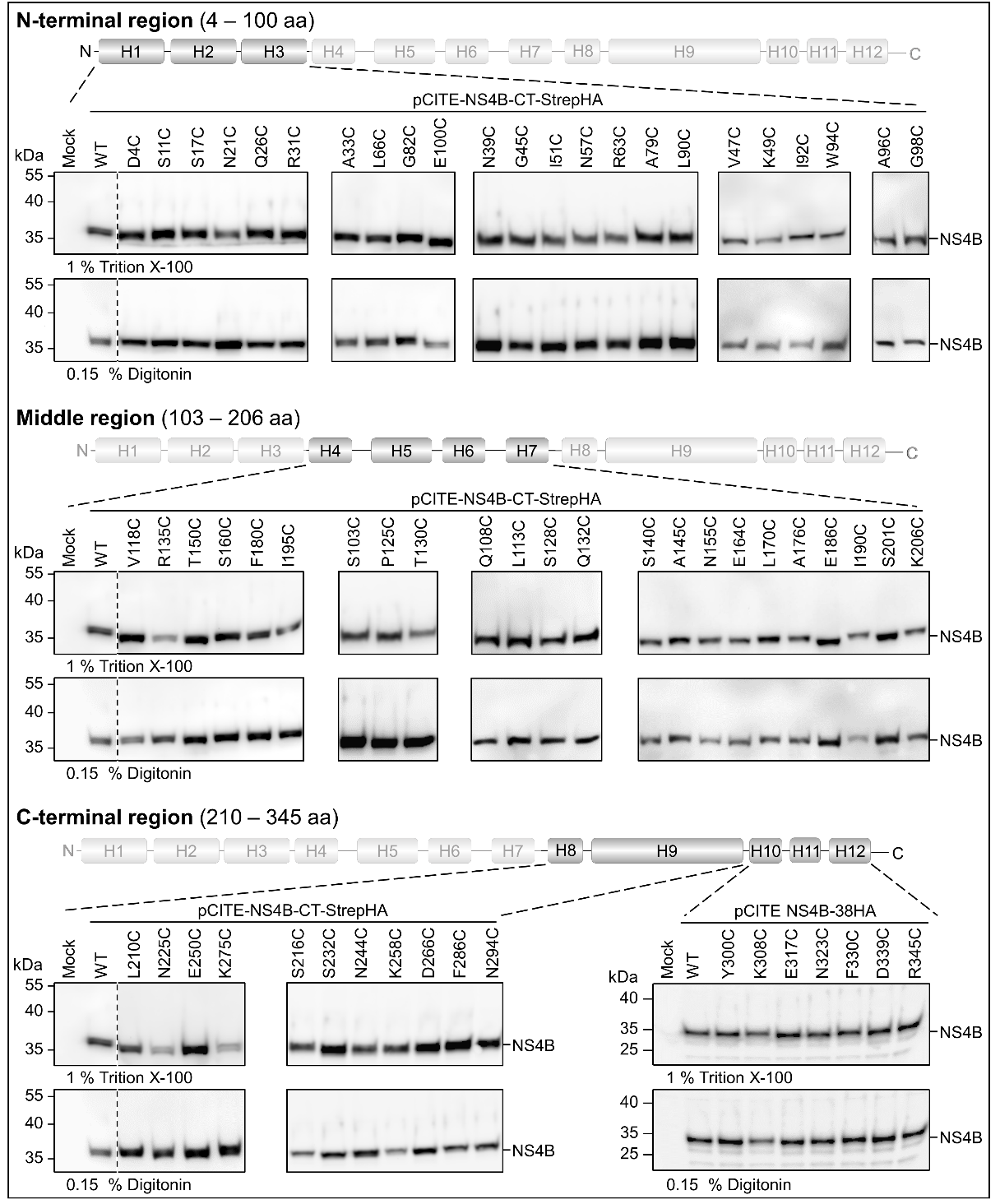


**Fig. S5) Western Blot analysis after SCAM of samples not treated with PEG-maleimide.** For detection an α-HA specific antibody was used. (Top panel) N-terminal region (4 – 100 aa), (middle panel), central region (103 – 206 aa) and (bottom panel) C-terminal region (210 – 345 aa) of NS4B. Treatment of samples (Triton X-100 or Digitonin) is indicated below each blot. Samples shown were produced in different experiments. Samples shown within one box are derived from the same blot. Mock and wild type controls are from one representative Western Blot. The original blots including the respective mock and wild type samples are provided in S7. Reference protein masses (kDa) are indicated on the left and detected signals are described on the right.

**Table S6) Quantitative analysis of Western Blots after SCAM.** For Digitonin-lysed and PEG-maleimide treated samples the mean signal intensity for NS4B and NS4B* was calculated with Image J and the ratio NS4B*/NS4B determined. For samples without visible NS4B* signal the same area used for the NS4B signal was measured at the expected height of the sample lane. Measured values were corrected for the background signal of the blot membrane. The ratios were normalized to the sample R345C (expected to be cytoplasmic) and color-coded according to arbitrary cut-off limits for accessibility (red: < 15 %, inaccessible; yellow 15 – 30 %, partly accessible; > 30 %, accessible). NS4B: non-modified NS4B, NS4B*: PEG-maleimide modified NS4B; RU: relative units.

| **AA** | **Protein** | **Area [cm^2^]** | **Signal [RU]** | **Ratio NS4B*/NS4B**  **(normalized)** | **AA** | **Protein** | **Area [cm^2^]** | **Signal [RU]** | **Ratio NS4B*/NS4B**  **(normalized)** |
| --- | --- | --- | --- | --- | --- | --- | --- | --- | --- |
| **D4** | NS4B | 0.057 | 48398 | 43.83 % | **S140** | NS4B | 0.036 | 43816 | 6.66 % |
|  | NS4B* | 0.047 | 16559 |  |  | NS4B* | 0.036 | 1765 |  |
| **S11** | NS4B | 0.058 | 46403 | 72.25 % | **A145** | NS4B | 0.039 | 49671 | 12.19 % |
|  | NS4B* | 0.044 | 33627 |  |  | NS4B* | 0.039 | 3789 |  |
| **S17** | NS4B | 0.051 | 41596 | 87.87 % | **T150** | NS4B | 0.056 | 52690 | 4.48 % |
|  | NS4B* | 0.045 | 43479 |  |  | NS4B* | 0.056 | 1409 |  |
| **N21** | NS4B | 0.048 | 59550 | 85.06 % | **N155** | NS4B | 0.035 | 33167 | 76.41 % |
|  | NS4B* | 0.046 | 58309 |  |  | NS4B* | 0.057 | 26531 |  |
| **Q26** | NS4B | 0.048 | 47448 | 77.88 % | **S160** | NS4B | 0.048 | 54288 | 67.57 % |
|  | NS4B* | 0.048 | 39288 |  |  | NS4B* | 0.049 | 35148 |  |
| **R31** | NS4B | 0.043 | 44496 | 77.82 % | **E164** | NS4B | 0.036 | 46111 | 43.17 % |
|  | NS4B* | 0.036 | 36790 |  |  | NS4B* | 0.045 | 15457 |  |
| **A33** | NS4B | 0.039 | 32571 | 81.47 % | **L170** | NS4B | 0.033 | 41728 | 40.10 % |
|  | NS4B* | 0.038 | 29334 |  |  | NS4B* | 0.051 | 12692 |  |
| **N39** | NS4B | 0.041 | 57070 | 38.57 % | **A176** | NS4B | 0.039 | 51676 | 25.94 % |
|  | NS4B* | 0.040 | 16505 |  |  | NS4B* | 0.044 | 9181 |  |
| **G45** | NS4B | 0.036 | 32506 | 86.56 % | **F180** | NS4B | 0.052 | 47353 | 38.82 % |
|  | NS4B* | 0.035 | 32956 |  |  | NS4B* | 0.060 | 13810 |  |
| **V47** | NS4B | 0.054 | 60384 | 13.83 % | **E186** | NS4B | 0.059 | 48903 | 7.63 % |
|  | NS4B* | 0.048 | 5280 |  |  | NS4B* | 0.059 | 2271 |  |
| **K49** | NS4B | 0.058 | 40639 | 81.88 % | **I190** | NS4B | 0.040 | 21795 | 10.00 % |
|  | NS4B* | 0.051 | 36950 |  |  | NS4B* | 0.040 | 1346 |  |
| **I51** | NS4B | 0.043 | 58590 | 5.54 % | **I195** | NS4B | 0.047 | 47575 | 1.74 % |
|  | NS4B* | 0.043 | 1952 |  |  | NS4B* | 0.047 | 485 |  |
| **N57** | NS4B | 0.040 | 54961 | 15.89 % | **S201** | NS4B | 0.046 | 39527 | 9.71 % |
|  | NS4B* | 0.038 | 5595 |  |  | NS4B* | 0.046 | 2367 |  |
| **R63** | NS4B | 0.044 | 40045 | 4.97 % | **K206** | NS4B | 0.034 | 28265 | 64.32 % |
|  | NS4B* | 0.044 | 1193 |  |  | NS4B* | 0.055 | 16894 |  |
| **L66** | NS4B | 0.043 | 45891 | 8.92 % | **L210** | NS4B | 0.050 | 44911 | 23.64 % |
|  | NS4B* | 0.043 | 2512 |  |  | NS4B* | 0.050 | 7157 |  |
| **A78** | NS4B | 0.044 | 55260 | 11.41 % | **S216** | NS4B | 0.036 | 10551 | 3.35 % |
|  | NS4B* | 0.044 | 3929 |  |  | NS4B* | 0.036 | 209 |  |
| **G82** | NS4B | 0.044 | 53410 | 13.92 % | **N225** | NS4B | 0.047 | 41159 | 21.52 % |
|  | NS4B* | 0.033 | 4707 |  |  | NS4B* | 0.044 | 5889 |  |
| **L90** | NS4B | 0.053 | 60692 | 1.14 % | **S232** | NS4B | 0.036 | 17922 | 1.63 % |
|  | NS4B* | 0.053 | 407 |  |  | NS4B* | 0.036 | 172 |  |
| **I92** | NS4B | 0.055 | 62837 | 11.25 % | **N244** | NS4B | 0.048 | 12585 | 20.65 % |
|  | NS4B* | 0.044 | 4400 |  |  | NS4B* | 0.050 | 1717 |  |
| **W94** | NS4B | 0.054 | 62298 | 2.88 % | **E250** | NS4B | 0.045 | 31179 | 85.68 % |
|  | NS4B* | 0.045 | 1061 |  |  | NS4B* | 0.086 | 30971 |  |
| **A96** | NS4B | 0.041 | 59872 | 0.35 % | **K258** | NS4B | 0.038 | 4868 | 63.47 % |
|  | NS4B* | 0.041 | 121 |  |  | NS4B* | 0.051 | 2849 |  |
| **G98** | NS4B | 0.054 | 61469 | 3.81 % | **D266** | NS4B | 0.044 | 10736 | 62.64 % |
|  | NS4B* | 0.074 | 1393 |  |  | NS4B* | 0.044 | 6152 |  |
| **E100** | NS4B | 0.052 | 16781 | 116.76 % | **K275** | NS4B | 0.058 | 36602 | 46.30 % |
|  | NS4B* | 0.053 | 35510 |  |  | NS4B* | 0.068 | 13487 |  |
| **S103** | NS4B | 0.044 | 49034 | 55.14 % | **F286** | NS4B | 0.044 | 8605 | 2.87 % |
|  | NS4B* | 0.047 | 23149 |  |  | NS4B* | 0.046 | 146 |  |
| **Q108** | NS4B | 0.084 | 59735 | 4.08 % | **N294** | NS4B | 0.032 | 10460 | 4.59 % |
|  | NS4B* | 0.084 | 1451 |  |  | NS4B* | 0.043 | 287 |  |
| **L113** | NS4B | 0.059 | 60750 | 1.64 % | **Y300** | NS4B | 0.039 | 63227 | 9.67 % |
|  | NS4B* | 0.059 | 586 |  |  | NS4B* | 0.045 | 3767 |  |
| **V118** | NS4B | 0.054 | 49307 | 1.43 % | **K308** | NS4B | 0.035 | 50209 | 9.58 % |
|  | NS4B* | 0.052 | 412 |  |  | NS4B* | 0.028 | 2962 |  |
| **P125** | NS4B | 0.037 | 45854 | 50.84 % | **E317** | NS4B | 0.026 | 29301 | 92.69 % |
|  | NS4B* | 0.037 | 19252 |  |  | NS4B* | 0.039 | 34269 |  |
| **S128** | NS4B | 0.043 | 57459 | 65.13 % | **N323** | NS4B | 0.030 | 58756 | 7.72 % |
|  | NS4B* | 0.051 | 35034 |  |  | NS4B* | 0.030 | 2764 |  |
| **T130** | NS4B | 0.039 | 48779 | 25.26 % | **F330** | NS4B | 0.036 | 60005 | 12.10 % |
|  | NS4B* | 0.041 | 8399 |  |  | NS4B* | 0.036 | 4542 |  |
| **Q132** | NS4B | 0.049 | 59448 | 23.78 % | **D339** | NS4B | 0.029 | 35359 | 83.20 % |
|  | NS4B* | 0.047 | 9539 |  |  | NS4B* | 0.028 | 33150 |  |
| **R135** | NS4B | 0.049 | 46724 | 6.38 % | **R345** | NS4B | 0.026 | 30798 | 100.00 % |
|  | NS4B* | 0.049 | 1801 |  |  | NS4B* | 0.025 | 42811 |  |


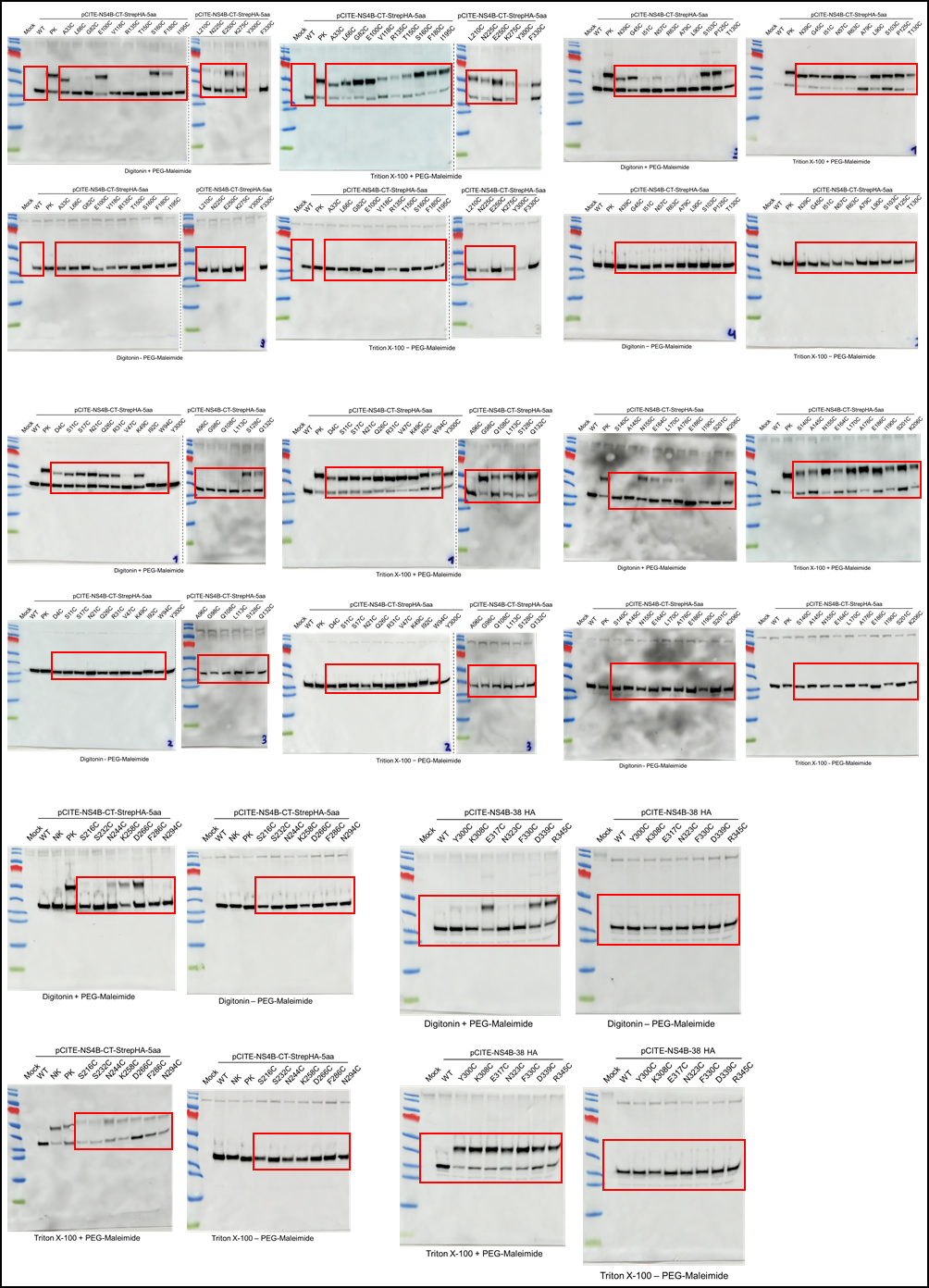


**Figure S7) Additional data referring to figures 4C-E and S5.** The blot area depicted in the respective figures are highlighted.
